# Culture-Independent Survey of Thermophilic Microbial Communities of the North Caucasus

**DOI:** 10.3390/biology10121352

**Published:** 2021-12-20

**Authors:** Stepan V. Toshchakov, Anna O. Izotova, Elizaveta N. Vinogradova, Gennady S. Kachmazov, Albina Y. Tuaeva, Vladimir T. Abaev, Martha A. Evteeva, Natalia M. Gunitseva, Aleksei A. Korzhenkov, Alexander G. Elcheninov, Maxim V. Patrushev, Ilya V. Kublanov

**Affiliations:** 1 Kurchatov Center for Genome Research, National Research Center “Kurchatov Institute”, Ac. Kurchatov Square, 1, Moscow 123098, Russia; Izotova_AO@nrcki.ru (A.O.I.); Vinogradova_EN@nrcki.ru (E.N.V.); Evteeva_MA@nrcki.ru (M.A.E.); Gunitseva_NM@nrcki.ru (N.M.G.); Korzhenkov_AA@nrcki.ru (A.A.K.); Patrushev_MV@nrcki.ru (M.V.P.); 2 Faculty of Biology, Lomonosov Moscow State University, 1-12 Leninskie Gory, Moscow 119991, Russia; 3 Faculty of Chemistry, Biology and Biotechnology, North Ossetian State University Named after K.L. Khetagurov, Vatutina str., 44-46, Vladikavkaz 362025, Russia; kgssogutfi@yandex.ru (G.S.K.); hampazero@mail.ru (V.T.A.); 4 National Research Center Kurchatov Institute-GOSNIIGENETIKA, 1st Dorozhny Pr., 1, Moscow 117545, Russia; tuaeva_97@bk.ru; 5 Winogradsky Institute of Microbiology, Research Center of Biotechnology RAS, 60-let Oktyzbrya Av., 7/2, Moscow 119071, Russia; elcheninov.ag@gmail.com (A.G.E.); kublanov.ilya@gmail.com (I.V.K.)

**Keywords:** thermophiles, North Caucasus, North Ossetia, deep thermal aquifer, hot spring, metabarcoding, 16S, extremophiles, extreme environments

## Abstract

**Simple Summary:**

The Republic of North Ossetia-Alania, located in the southern part of the North Caucasus, possess a number of hydrothermal habitats, including both subterranean thermal reservoirs and terrestrial hot springs. At the same time, reports on microbiology of numerous geothermal sites are rather scarce for the whole North Caucasus region. In this paper, we report on the first culture-independent metabarcoding study of thermal habitats in the North Caucasus, coupled with a chemical analysis of the elemental composition of water. The results of this work include the conclusions regarding key metabolic characteristics of these habitats as well as detection of few but abundant deep lineages of uncultivated microorganisms which could be regarded as endemic. This study may represent a first step in closing the knowledge gap in extremophilic microbial communities of the North Caucasus.

**Abstract:**

The Greater Caucasus is a part of seismically active Alpine–Himalayan orogenic belt and has been a center of significant volcanic activity during the Quaternary period. That led to the formation of the number of hydrothermal habitats, including subterranean thermal aquifers and surface hot springs. However, there are only a limited number of scientific works reporting on the microbial communities of these habitats. Moreover, all these reports concern only studies of specific microbial taxa, carried out using classical cultivation approaches. In this work, we present first culture-independent study of hydrotherms in the Republic of North Ossetia-Alania, located in the southern part of the North Caucasus. Using 16S metabarcoding, we analyzed the composition of the microbial communities of two subterranean thermal aquifers and terrestrial hot springs of the Karmadon valley. Analysis of correlations between the chemical composition of water and the representation of key taxa allowed us to identify the key factors determining the formation of microbial communities. In addition, we were able to identify a significant number of highly abundant deep phylogenetic lineages. Our study represents a first glance on the thermophilic microbial communities of the North Caucasus and may serve as a basis for further microbiological studies of the extreme habitats of this region.

## 1. Introduction

Geothermal areas are unique habitats in which extremophilic microbial realms actively function, carrying out diverse metabolic processes. Although temperature and pH are considered to be the main factors that shape the structure of thermophilic microbial communities [1], chemical composition of thermal water and sediments also play an important role, defining the energy flow and biogeochemical cycles, and driving the microbial communities [2,3,4]. Although its relevance is a subject of scientific debates, geographical location is another factor determining the microbial communities of hot springs with similar parameters [5]. The commonly accepted hypothesis “everything is everywhere” may not be entirely true for microorganisms living in isolated habitats like caves, high altitude areas, or underground reservoirs, as opposed to environments mixed actively by air or water currents [6]. In this context, extremophilic microorganisms, which are extremely resistant to stress, but grow only in harsh environmental conditions, are of particular interest. Despite the fact that active thermophilic microorganisms were found hundreds and thousands of kilometers away from hydrothermal sites [7,8], indicating that at least some of them can bear the cold and oxygenated environments for a long time, it seems that the majority of thermophilic cells have a considerably lower probability to survive under conditions of dehydration (on a surface) or at low temperatures [9]. This is heightened by the fact that the vast majority of true thermophiles (T_opt_ > 60 °C) do not possess spores. Therefore, while microbial communities, which inhabit distantly located but hydrochemically similar hot springs are usually similar on the level of higher taxa, some springs might be inhabited by populations, which include a significant proportion of endemic microorganisms [10].

Caucasus is a mountainous region between the Black and Caspian seas. The North Caucasus located north of the Greater Caucasus Mountain Range is enriched in both terrestrial and subterranean thermal habitats [11,12]. Microbial communities inhabiting these ecotopes have been little studied. Currently, there are only few reports about microbial diversity in these habitats based on classical microbiological approaches [13,14,15]. According to our knowledge, metagenomic or metabarcoding-based surveys of North Caucasian thermal environments had not been reported so far [16].

Present work is focused on thermal habitats of the Republic of North Ossetia-Alania located in the southern part of the North Caucasus and separated from Georgia and South Ossetia by Greater Caucasus Range. Geothermal habitats in the region include both subterranean thermal reservoirs and terrestrial hot springs [17]. These habitats cannot be characterized by extreme temperatures, because the maximal temperature, which was measured there, was 55 °C. However, their level of isolation from other well-studied thermal biotopes suggests that their microbial communities might possess novel and uncultured taxonomic lineages. Moreover, the diversity of the physicochemical parameters of these habitats suggests that thermophilic microbial communities may be highly variable in their composition.

Here, we report the first culture-independent microbiological survey of the North Ossetia-Alania hot springs, which was conducted using high-throughput metabarcoding analysis coupled with analysis of thermal water chemical composition. For each hydrothermal habitat, we analyzed the community structure of both water and sediments. To achieve better resolution of water microbial communities we implemented size fractionation sequential filtering—a common strategy, applied in marine microbiological studies, both to detect small-sized ‘microbial dark matter’ and to identify the microorganisms, associated with particulate organic matter [18,19].

## 2. Materials and Methods

### 2.1. Sample Collection

**Figure 1:**
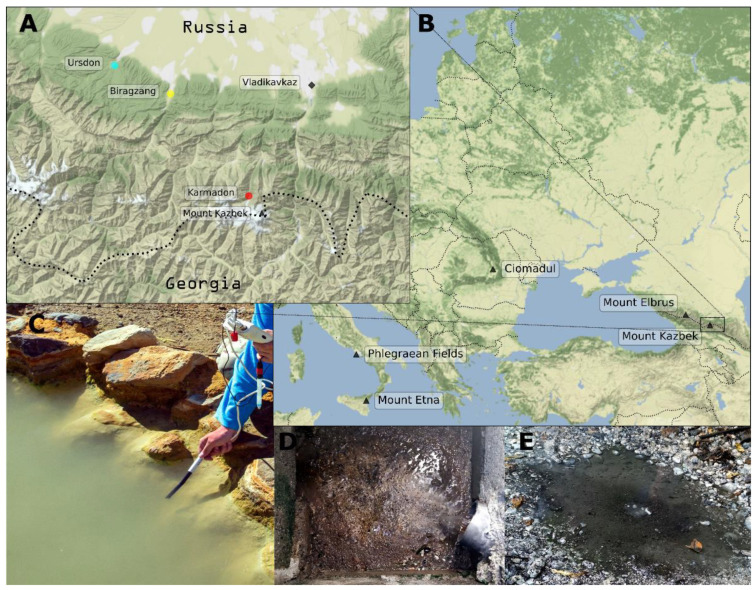
Map and photographs of sampling sites of the Republic of North Ossetia-Alania. (**A**) Map at region scale; (**B**) map at continent scale, active volcanoes indicated by black triangles; (**C**) part of natural travertine-formed thermal bath at Karmadon spring #4135; (**D**) sediment under the thermal water outlet at Biragzang deep well; (**E**) hydrosulfide-rich puddle formed as a result of thermal water pipe seepage near the Ursdon well outlet.

Samples of water and sediments were collected from three geothermal sites located in North Ossetia (Figure 1, Table 1). For DNA extraction, 5–10 L of water samples were sequentially filtered through 0.45 and 0.22 µm Sterivex^TM^ filters (Merck, Darmstadt, Germany) and fixed with RNAlater (ThermoFischer Scientific, Waltham, MA, USA). Sediments and microbial mats were sampled in 5 mL Eppendorf tubes; excess water was removed, and tubes were filled with RNAlater. Thus, four samples of sediments, one microbial mat, and four samples of water were taken. All samples were kept at 4 °C for a week during transportation to the research lab in Moscow.

### 2.2. Analysis of Water Physicochemical Parameters and Element Composition

Temperature, pH, and redox potential were measured on site with the help of SevenGo™ portable device (Mettler-Toledo, Greifensee, Switzerland), according to manufacturer’s instructions. Water samples for elements analysis were taken in 50 mL Falcon tubes and stored at +4 °C until analyzed.

Elemental analysis of thermal water was performed at Institute for Problems of Microelectronics Technology and High-Purity Materials RAS by mass spectrometry and atomic emission spectrometry with inductively coupled plasma, using Perkin Elmer ELAN model DRC-e mass spectrometer. The power of the plasma generator was 1250 W, the voltage at the detector was 1400 W. The atomizing argon flow was 0.91–0.96 L/min, the auxiliary argon flow was 1.15 L/min, and the argon flow through the orifice was 15 L/min. Measured values and limits of detection are presented in Table 2. In total, four samples of thermal water were analyzed for element composition.

### 2.3. DNA Isolation

For DNA extraction Sterivex filters were taken out from the cover and cut into several pieces with sterile scissors. The filter sections were placed into PowerBeads tubes from the Qiagen PowerLyzer PowerSoil kit (Qiagen, Hilden, Germany). One 5 mm stainless steel bead (Qiagen, Germany) was added into each PowerBead tube to improve bead-beating efficiency. Other steps were performed with Qiagen PowerLyzer PowerSoil kit (Qiagen, Germany) according to manufacturer’s instructions. Sediments were isolated with Qiagen PowerLyzer PowerSoil kit according to manufacturer’s instructions without any modifications. Negative control was run with sterile water instead of biomass with each processed sample batch. Quantification of extracted environmental DNA was performed with a Qubit fluorometer.

### 2.4. High Throughput 16S Community Profiling

Amplicon libraries for V4 hypervariable region of 16S rRNA gene were prepared using a two-step PCR strategy [20]. Each specimen of extracted environmental DNA and two PCR negative controls were run in two PCR-replicates. The first round of PCR was performed using qPCRmix-HS SYBR (Evrogen, Russia) with the following primers at 0.25 µM concentration: V4_515F TCGTCGGCAGCGTCAGATGTGTATAAGAGACAG [NN] GTGBCAGCMGCCGCGGTAA and V4_805R GTCTCGTGGGCTCGGAGATGTGTATAAGAGACAG [NN] GACTACNVGGGTMTCTAATCC where the first part corresponded to partial Illumina TruSeq adapter, [NN] corresponded to 1–3 nt degenerate heterogeneity spacer and the last part corresponded to 515F [21] and Pro-mod-805R [22] primers, respectively (Supplementary Table S7). Amplification was performed by CFX96 Touch Real-Time PCR Detection System (Bio-Rad, USA). Cycling parameters were the same as described before [23]. Depending on the amplification curve the amplification mix was diluted 4–8 times and used as a matrix for the second PCR. The second PCR was performed with ScreenMix-HS (Evrogen, Russia) using the following primers at 0.5 µM concentration: R1TM AATGATACGGCGACCACCGAGATCTACACA XXXXXX CGTCGGCAGCGTC and R2TM CAAGCAGAAGACGGCATACGAGAT XXXXXX GTCTCGTGGGCTCGG, where the first part corresponded to P5 or P7 Illumina flowcell oligonucleotides, XXXXXX corresponded to 6 nt index sequences and the last part corresponded to partial Illumina TruSeq adapters, annealing to the tail of first PCR primers (Supplementary Table S7). Amplification was performed by Veriti Thermal Cycler (Applied Biosystems, Bedford, MA, USA) using cycling parameters, described previously [23]. Resulting libraries were checked on the agarose gel and pooled equimolarly. Final pool was purified with QIAquick Gel Extraction Kit (Qiagen, Germany) according to manufacturer’s protocols.

Sequencing was performed using MiSeq™ Personal Sequencing System (Illumina, San Diego, CA, USA) using 156 bp paired-end reads. ‘Trim reads’ tool of CLC Genomics Workbench 20.0 (Qiagen, Germany) was used quality filtering and removing of residues, corresponding to 515F and Pro-mod-805R primers. Demultiplexing was performed with the deML package with default parameters [24]. The number of read pairs obtained from each replicate was in the range of 13–32 thousand per sample (Supplementary Table S1).

### 2.5. Data Analysis

High quality read pairs were used as an input for DADA2 pipeline [25], which was run according to DADA2 tutorial (https://benjjneb.github.io/dada2/tutorial.html, accessed on 19 November 2021), except the read truncation step, which was not performed, since it was unnecessary due to the stringent read quality filtering during data pre-treatment. Taxonomy of amplified sequence variants (ASVs) was assigned with a naive Bayesian classifier using Silva138 16S rRNA gene database [26]. Obtained ASV reference sequences, sample metadata, abundance, and taxonomy tables were imported in the phyloseq package [27], and all further operations were performed with the phyloseq object. Decontamination of amplicon data was performed with the *decontam* R package using “prevalence” contaminant identification method with default parameters [28]. Alpha and beta diversity analysis and visualizations were made with *phyloseq* [27], *vegan* [29], and *microbiome* [30] R packages. Rarefaction curve analysis was performed with the *iNEXT* package [31]. Canonical correspondence analysis and fitting of environmental variables were performed using *cca* and *envfit* functions of *vegan* R package with default parameters. Analysis of correlations between microbial genera and element composition was performed with *associate* function of *microbiome* R package [30] using Spearman’s rank-order correlation method.

## 3. Results

### 3.1. Description and Physicochemical Parameters of Sampling Sites

Biragzang groundwater deposit located on the right bank of the Ardon River, which is 35 km to the west from Vladikavkaz, was studied by detailed geological examination in early 90s (Figure 1). Drilling of well 1BT (2370 m deep) resulted in discharge of 53 °C sodium chloride-bicarbonate thermal mineral water. During next two decades the temperature and mineralization of water decreased from 53 to 50 °C and from 2.1 to 1.1 g/L, respectively [32]. The content of bicarbonate anions in 2017 was 56.7–74.5 mg/L, sodium and potassium cations was 10 mg/L. The content of silicic acid was 33–35 mg/L. Since 2006, the well was used as a thermal water source for the local thermal spa. At the moment of sampling, temperature of the water was 48 °C and pH was 8.8 (Table 1). Redox state of discharging water was slightly reduced with Eh in the range of −20 to 0 mV. Analysis of elemental chemical composition showed significant enrichment of molybdenum (15.6 µg/L), compared to other thermal waters, sampled during this study. Additionally, several rare elements as cadmium, aluminum, vanadium, and antimonium which have not been detected in other waters, were found in significant amounts (Table 2).

Ursdon sampling site belongs to Kora mineral water deposit located in the valley of the Ursdon river, which is 50 km to the west from Vladikavkaz, at the height of 600–700 m above sea level (Figure 1, Table 1). The well 3e, which was used for the sampling is 1530 m deep. According to the reports of local authorities, the water temperature, pH, and mineralization of discharge are 56 °C, 7.3 and 7.5 g/L, respectively. The water is enriched by hydrogen sulfide as well as calcium and magnesium ions. At sampling time, the water temperature at the outlet was 47 °C, pH was 7.0 (Table 1). Ursdon waters had the highest concentration of sulfur, calcium, and magnesium among the samples (867, 651, and 171 mg/L, respectively).

The Upper Karmadon hot springs are located 6 km north-northwest from the summit of Mount Kazbek and 35 km southwest from Vladikavkaz. The springs’ water is strongly enriched with bicarbonate anions, resulting in the formation of travertine baths used by the locals and tourists for balneo therapeutic purposes [33]. According to the first systematic work carried out in the 1960s, the Upper Karmadon geothermal field has more than 50 thermal water outlets [34]. Water and sediments were sampled from two outlets, located in the range of 20–30 m from each other. Temperature of the sampled water was in the range of 52–55 °C, pH was 6.1. Chemical analysis supported previous observations of the high level of mineralization in Karmadon waters [34]. Compared to subterranean habitats water was enriched by sodium, boron, silica, potassium, iron, manganese, strontium, and numerous rare elements (Table 2; Supplementary Figure S1).

### 3.2. Analysis of Prokaryote Diversity Using 16S rRNA Profiling

In total, 13 samples of environmental DNA were analyzed by 16S rRNA metabarcoding. Microbial diversity of samples was assessed using 515F [21] and Pro-mod-805R [22] primers to V4 hypervariable region of 16S rRNA gene. PCR was performed in two replicates. After read filtering, merging and chimera removal performed by DADA2 pipeline [25], 476 thousand reads were used for the analysis (Supplementary Table S1). After in silico decontamination of the resulting ASV table, performed by *decontam* package [28] 1507 ASVs, corresponding to 676 genera were obtained. Repeatability of PCR replicates, checked by analysis of NMDS ordination, based on Bray–Curtis distances (Supplementary Figure S2), allowed merging replicates for further analysis. Final rarefied dataset was represented by 13 samples of 24,935 reads each.

**Figure 2:**
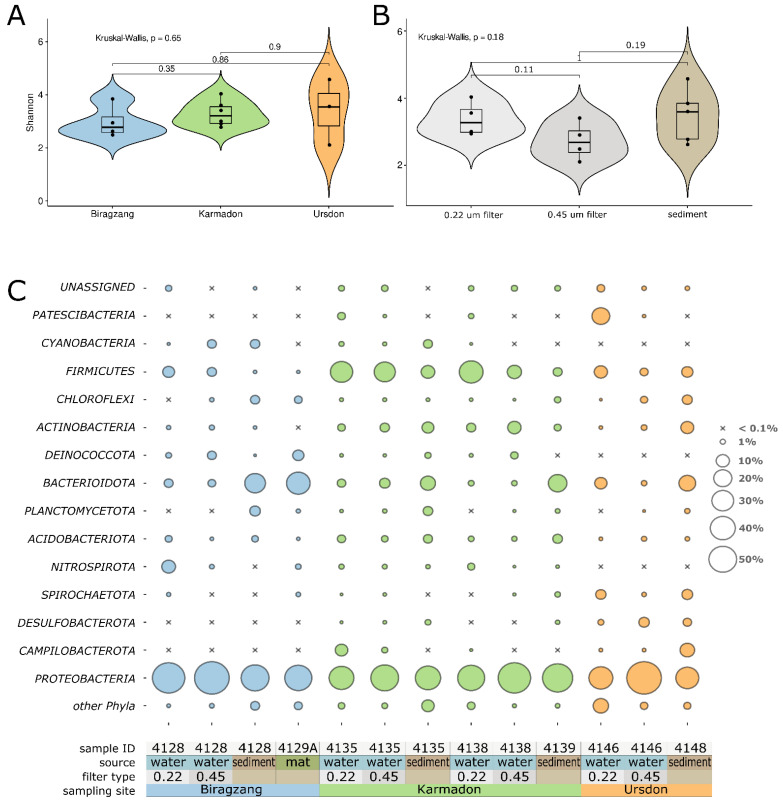
Alpha diversity and taxonomic composition of thermophilic microbial communities of North Ossetia-Alania: (**A**) violin plots of Shannon indexes compared between sampling sites; (**B**) violin plots of Shannon indexes compared between community fractions, retained 0.22 µm, 0.45 µm and sediments; (**C**) bubble plot of phyla abundances in each sample.

ASV rarefaction curves built with iNEXT R package [31] reached the saturation approximately at 20,000 reads for all sequenced samples (Supplementary Figure S3), indicating the sufficiency of sequencing depth. Analysis of alpha-diversity metrics of rarefied dataset showed tangible variation between different probes (Supplementary Table S2); however, statistical comparison of sample groups, formed by either sampling field or treatment type, has not revealed significant differences (Figure 2A,B). The highest mean value of detected ASVs was determined for the sediment under the discharge of neutral Ursdon well (322 ASVs); however, that might be explained by the mix of thermophilic microorganisms with the soil-derived community (*Rhizorhapis* sp., *Nocardioides* sp., *Arenimonas* sp., etc.). Alpha diversity metrics of Karmadon and Biragzang sediments samples were not only lower in comparison with Ursdon samples but also with respective Karmadon and Biragzang water samples. Alpha diversity metrics of the fraction of microbial community, retained on 0.22 µm filters were slightly higher than those of 0.45 µm fraction (Supplementary Table S2). This observation is being in line with significant abundances of ultrasmall microbial forms (Patescibacteria) in 0.22 µm-filtered samples, which obviously pass through 0.45 µm filter.

**Figure 3:**
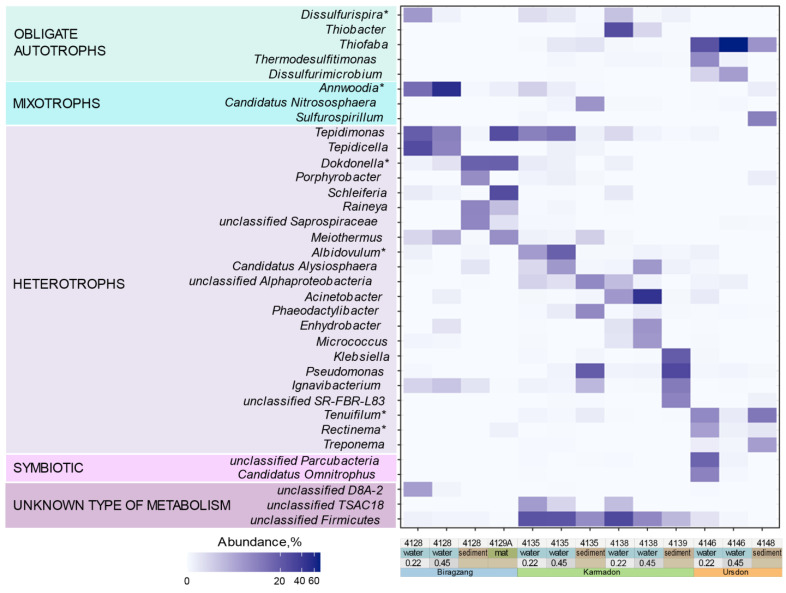
Abundance heatmap of prokaryotic genera, comprising more than 5% at least in one of the samples. Microbial taxa are grouped by metabolic properties of closest cultivated relatives. Asterisks near the taxa names indicate that taxonomy, assigned by DADA-2 Bayesian classifier was further refined by BLAST search against NCBI type material 16S rRNA database.

All the analyzed communities showed clear dominance of Bacteria which make up more than 99% of the microbial population. Archaea were represented in significant amounts (>1%) only by Ca. *Nitrososphaera* in the sediments of the Karmadon hot spring (6.22%) and by unclassified Hadarchaeales archaeon in the sediments under the discharge of Ursdon thermal well (1.36%). Bacterial amplicon tags were assigned to 43 different phyla, 20 of which were present in more than 1% of total microorganisms (at least in one of the samples). In all samples, Proteobacteria was the dominant phylum constituting 33.62 to 80.42% of the communities (Figure 2C). In most cases Proteobacteria was represented by Gammaproteobacteria. Particle-associated fraction (retained on 0.45 µm filter) of Biragzang water was dominated by two ASVs assigned to Hydrogenophilaceae representatives (51.16% totally). Despite not having been classified at lower taxonomic level by DADA2, BLAST search using these ASVs as queries against NCBI 16S rRNA genes of pure cultures database and showed 96–97% identity to *Annwoodia aquaesulis* (Supplementary Table S5) [35]. We assume that the dominance of these ASVs in 0.45 µm compared to 0.22 µm fractions (Figure 3) is due to the capability of *Annwoodia* to associate with mineral particles, which was documented in several reports for closely related *Thiobacillus ferrooxidans* [36,37]. In turn, the dominating microorganisms from Biragzang thermal water, retained on 0.22 µm filter were two representatives of Comamonadaceae: *Tepidimonas* and *Tepidicella* (18.91 and 18.83%, respectively). In Ursdon waters, highly enriched by sulfur, the dominant gammaproteobacterial ASVs (60% of the 0.45 filter retained fraction) were classified as *Thiofaba* and showed 99.6% identity to *Thiofaba tepidiphila*, isolated from 45 °C hot spring in Japan [38]. In the terrestrial Karmadon hot springs, the Gammaproteobacteria were much more diverse and were represented by 64 different genera, 15 of which had more than 1% abundance at least in one of the samples. However, the structure of gammaproteobacterial community was different in the two Karmadon sampling sites. Site #4138 showed significant amounts of *Thiobacter* (up to 26.57% in 0.22 µm filter fraction), which was completely absent in site #4135 (Figure 3). #4135 water, in turn, was enriched by *Tepidimonas* (11.4% of total reads). Anthropogenic load of the Karmadon hot springs was reflected in the composition of sedimental and particle-associated samples, which showed high abundances of Enterobacterales and Pseudomonadales (Figure 3, Supplementary Figure S4).

Alphaproteobacteria were present in comparable amounts with Gammaproteobacteria in some sediment samples (Figure 2C). The sediment under the discharge of Biragzang thermal well contained 23.09% of alphaproteobacterial cells, prevailed by *Porphyrobacter* and *Sandaracinobacter* representatives. In turn, alphaproteobacteria from Ursdon well sediment (14.09% of total) were represented by a number of Sphingomonadaceae members constituting 8.09% of the total community. Alphaproteobacteria of the Karmadon springs, again, were more diverse and the most numerous genera were *Albidovulum, Candidatus Halyseosphaera, Iodidimonas* and uncultured Iodidimonadaceae-related bacteria, making up 17.89, 5.84, 1.96, and 6.7 percent of the community, respectively.

The second most abundant phylum after Proteobacteria was Firmicutes, reaching more than 30% of all microorganisms in several samples (Figure 2C). The most abundant ASV of this phylum (2.45–22.49%) was ASV0001, classified as unknown Firmicutes bacterium distantly related to uncultured TSAC18 lineage [39] and present mainly in the Karmadon springs water and sediments. Blast search against NCBI pure cultures database showed ASV0001 was 87.35–88.19% identical to Thermoanaerobacteraceae representatives. Search against the environmental NCBI database resulted in a single hit with more than 95% identity, which is a V4 amplicon OTU from the soil, sampled after volcanic eruption on Kasatochi Island, Alaska [40]. Search in a JGI environmental genome database resulted in three 95% identity hits from Dewar Creek (Canada) hot spring sediments (Supplementary Table S3). The second abundant genus of Firmicutes, detected in the Karmadon springs was *Carboxydocella*, known to be an obligate chemolithoautotroph capable of hydrogenogenic carboxydotrophy [41,42].

Subterranean thermal waters of Biragzang possessed substantial number (5.3% in 0.22 µm filter samples) of Firmicutes, belonging to uncultured class D8A-2, which is shown to be enriched in consortium performing methanogenic degradation of volatile fatty acids [43]. Blast search of Biragzang D8A-2-related ASVs revealed that the most closely related microorganisms were detected in microbial communities of hydrotherms, located at East Tuva Plateau, Russia [44]. Other major fractions of Firmicutes in Biragzang belonged to Thiobacterales and Ammonifexales orders (Supplementary Figure S4). Representatives of the latter also were in significant abundance in the particle-associated fraction of Ursdon water (7.66%).

Representatives of the phyla Bacteroidota and Ignavibacteriota comprised of 1.26% to 35.78% of the communities with *Ignavibacterium* sp. and *Melioribacter* sp. being the most dominant genera (Figure 2C). In addition, an uncultured lineage SR-FBR-L83 was detected in significant amounts (8.73%) in the sediment of the Karmadon spring #4139 (Figure 3). This lineage is reported to be abundant in communities of Greater Obsidian Pool Area of Yellowstone National Park [45], and, according to some reports, enriches in hydrocarbon-amended microcosms [46]. Flavobacteriales were represented by *Schleiferia*, being the major component (27.07%) of the microbial mat found on the edges of thermal water tap in Biragzang. Other Bacteroidota members were detected mostly in sediments and assigned to Cytophagales, Chitinophagales, and Bacteroidales orders (Supplementary Figure S4).

Among other taxa Actinobacteria showed significant abundances (2.36–10.37%) in the Karmadon springs (Figure 2C), represented by Micrococcales and uncultivated order CG2-30-50-142. Several orders of Acidobacteria, namely, uncultivated Subgroup2, Thermoanaerobaculales and Aminicenantales were also presented in the Karmadon hot springs (Supplementary Figure S4). Other phyla, detected in the Karmadon springs were Camplilobacteria (mostly *Sulfuricurvum*), Cyanobacteria (uncultivated *Obscuribacteraceae*), Planctomycetota (mostly *Thermogutta*, *Planctomicrobium,* and uncultured *Planctomyces* sp. SH-PL14), Deinococcota, and Nitrospirota (Figure 2C).

Microorganisms from Ursdon deep well and the Karmadon hot springs retained on 0.22 µm filter were enriched by ultrasmall microbial forms belonging to the phyla Elusimicrobiota and Patescibacteria. Elusimicrobia ASVs were related to free-living groundwater Lineage IV [47] and made up to 1.9% in 0.22 µm filter sample from #4139 spring. Blast search of the most abundant 16S V4 amplicon variant of Elusimicrobia (ASV0135) against NCBI nr database gave 99–100% identity hits to sequences EU037201 and KC711401 related to microbial communities of Indian hot spring microbial mat and mesothermal spring water column in Mexica, respectively. Abundances of Patescibacteria in the Karmadon springs were 3.00% and 1.23% for springs #4135 and #4139, respectively (Figure 2C).

Several microorganisms-driving processes could be predicted according to the information on the metabolism of the closest relatives of the highly abundant North Ossetia ASVs. Most abundant chemolithoautotrophs were *Annwoodia* and *Dissulfurispira* (Biragzang), *Thiofaba, Thermodesulfitimonas, Sulfurospirillum* and *Dissulfurirhabdus* (Ursdon), and *Thiobacter* (Karmadon). Organoheterotrophs, capable of degrading proteins and carbohydrates, were represented by microorganisms *Schleiferia, Dokdonella, Tepidicella, Raineya* (Biragzang), *Tepidimonas* (Biragzang, Karmadon), *Albidovulum* and *Iodidimonas* (Karmadon), capable of respiration, as well as *Tenuifilum, Treponema, Rectinema,* and uncultured Parcobacteria members, predicted to grow by fermentation. Heterotrophic *Ignavibacterium* and uncultured *Ignavibacteria* representatives (Biragzang, Karmadon) can grow in both modes: respiration and fermentation, depending on growth conditions (Supplementary Table S5).

### 3.3. Analysis of Correlations between the Structure of the Microbial Community and Environmental Factors

**Figure 4:**
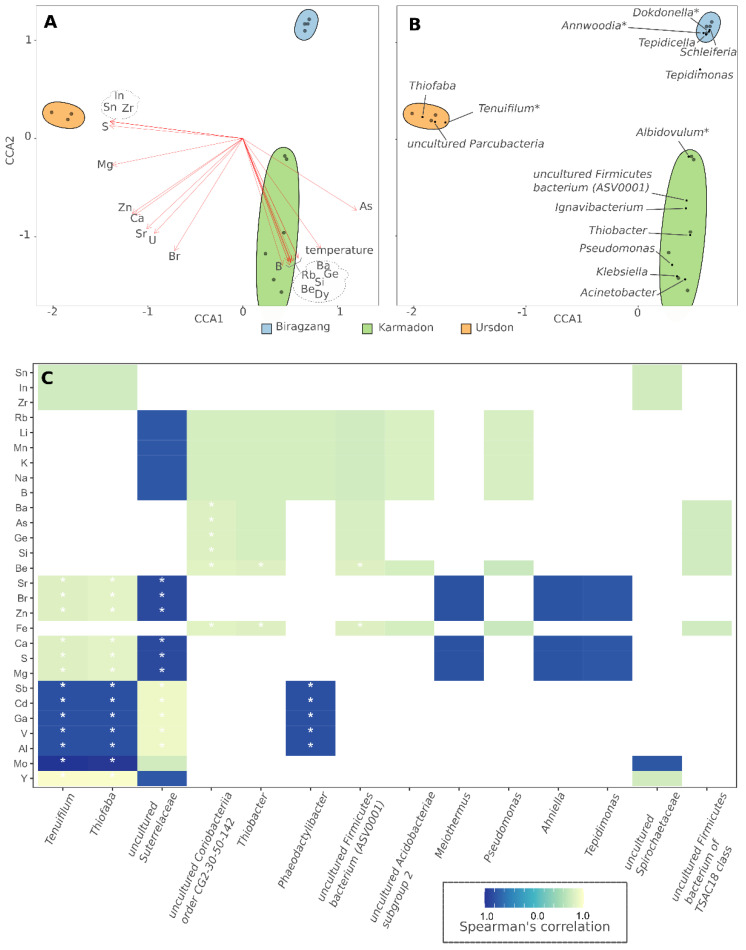
Correlation of microbial community composition with environmental parameters: (**A**) Environmental variables fitted to CCA plot by *envfit* function of vegan package; (**B**) Major taxa shown on CCA plot; (**C**) Heatmap of individual correlations of the most abundant taxa with elemental composition. Taxa-element pairs, which showed the correlation *p*-value less than 0.05, are marked with asterisks.

Canonical correspondence analysis (CCA) implemented by *vegan* R package was used to elucidate the key elements and environmental factors, influencing the composition of the microbial communities. For this purpose, only highly abundant genera (more than 10% at least in one sample) were included into the analysis. Resulting eigenvalues, reflecting the proportion of microbial community, which might be explained by ordination axes were 94.6% and 83.2% for CCA1 and CCA2, respectively. Communities, taken for the analysis from one sampling location demonstrated a high level of clustering (Figure 4). At least 20 elements including sulfur, bivalent metals, arsenic, and boron showed high levels of correlation (R2 value of *envfit* function > 0.9, with *p*-Value less than 0.01) with composition of microbial communities (Figure 4A,B; Supplementary Table S4). Sulfur appears to be the element with the most significant influence on the community composition, which explains the clear dominance of chemolithoautotrophic sulfur-oxidizing *Thiofaba* sp. in sulfur-rich Ursdon samples (Supplementary Table S5). The Karmadon springs, which are located in rocky areas characterized by relatively modern volcanic activity [48], were significantly enriched in rare elements, namely, rubidium, beryllium, dysprosium, barium, germanium, etc. Although the biological function of these elements is rather poor or unknown, some of them can be transported into prokaryotic cells and might perform an essential function in the extreme environment [49]. Arsenic was also enriched in the Karmadon springs and well correlated, according to CCA analysis, with *Albidovulum inexpectatum*, a representative of Rhodobacteraceae family, for members of which a positive correlation between their abundance and the presence of arsenate was demonstrated [50]. Biragzang water, characterized by low level of mineralization, did not demonstrate significant positive correlations of abundances of specific taxonomic groups with the composition of elements; however, it showed some enrichment of molybdenum, aluminum, and cadmium (Supplementary Table S4).

Individual correlations of the most abundant taxa with elemental composition, assessed by Spearman’s rank-order correlation analysis, showed that 14 genera significantly (*p*-Value < 0.05) correlated with element content in the environment. Thus, *Thiofaba* and *Tenuifilum* [51] correlated with sulfur and magnesium, as was also supported by CCA analysis. Interestingly, chemolitoautotrophic sulfur-oxidizing *Thiobacter* sp., abundant in the Karmadon springs, did not show correlation with sulfur, but strongly correlated with iron (*p* < 0.01; Figure 4C). Uncultured Firmicutes bacterium, corresponding to ASV0001, highly abundant in the Karmadon springs, demonstrated strong positive correlation with iron and beryllium (Figure 4C). Regarding the latter, it is unlikely that there is any connection between its presence and the structure of microorganism‘s community, rather its large amount is associated with iron.

## 4. Discussion

### 4.1. Diversity and Taxonomic Composition of Microbial Communities

Geothermal resources of the North Caucasus are considered to be important source of renewable hydrothermal energy [52], and therefore are actively studied from the geological perspective [11,53]. However, microbiological studies of these thermal habitats are rather scarce and focused on cultivation of few taxonomic groups [13,14,15]. In this study we performed the first culture-independent survey of thermal microbial communities of the Republic of North Ossetia, located in the southern part of the North Caucasus, adjacent to the Greater Caucasus mountain range. We surveyed two deep thermal wells of 1530 and 2370 m depth and surface hot springs located on the northern slope of Greater Caucasus range at an altitude of 2330 m. The latter was characterized by having the highest temperature (55 °C) and mineralization among studied habitats.

The majority of dominating taxa were moderate thermophiles which is in accordance with the water temperature. Relatively high abundance of Enterobacterales and Pseudomonadales in sediments and particle-associated fraction of the Karmadon springs was an exception, which can be explained by active use of these sources by tourists and the local population for balneological purposes. The increase in Pseudomonadales and Enterobacterales was also observed in post-bathing biomes of geothermal waters used for spa procedures [54].

Dominating taxa of the terrestrial Karmadon springs were presented by heterotrophic microorganisms in both water and sediment samples, which can be due to the presence of large amounts of allochtonic organic matter. In particular, the water was significantly enriched in obligate heterotrophs, utilizing sugars and amino acids, including *Albidovulum, Acinetobacter,* and *Moraxella*. Primary production in the Karmadon springs may be associated with a small number of detected autotrophic microorganisms, such as carboxydotrophic *Carboxydocella* [41] and sulfur/thiosulfate oxidizing *Thiobacter*, which uses CO_2_ as a sole carbon source [55]. It should be also noted that in the Karmadon springs we detected significant abundances of uncultivated taxa, in particular, dominating, but yet uncultured Firmicutes bacteria, corresponded to ASV0001 and ASV_0084 (up to 28.8% and 5.5%, respectively) implying the possibility that these bacteria may be also involved in primary production.

The overall content of autotrophic organisms in waters of subterranean thermal aquifers seemed to be higher than for terrestrial springs. 0.45 µm filter fraction of Biragzang deep well water showed clear dominance of ASV_0003 and ASV_0020, both of which corresponded to uncultured *Hydrogenophilaceae* representatives, close to *Annwoodia* sp., which is facultative autotroph, and able to utilize carbon dioxide or bicarbonate anion as the carbon source. The high amount of these microorganisms retained on a 0.45 µm filter might be explained by the supposition that they are associated with particles of carbonate rock through which the Biragzang groundwater flows. In turn, 0.22 µm filter fraction was dominated by two obligate heterotrophs, *Tepidicella xavieri* [56] and *Tepidimonas taiwanensis* [57], which might be explained by assuming these microorganisms came from organic-rich subsurface biosphere.

In turn, both fractions of Ursdon thermal water showed clear dominance of autotrophic organisms, implying they are from the rockier environment, which is being in accordance with the geography and/or depths of these two wells: Biragzang well is almost 800 m deeper (2370 vs. 1530 m) and might reach deeper, organic-rich layers.

Alpha diversity indexes of the terrestrial Karmadon hot springs roughly corresponded to diversity indices reported for New Zealand hot springs with similar temperature and pH parameters: 40–50 °C and circumneutral pH values [1]. In turn, values, determined for subsurface thermal habitats (Biragzang and Ursdon) were much higher than those reported for deep subsurface aquifers in Siberia and Poland, similar in temperature and pH [58,59]. However, those differences can be explained rather by OTU generation method (ASV vs. 97% sequence clustering), rather than on objective differences in diversity of microbial communities.

Overall, in all studied habitats, Bacteria domain significantly outnumbered Archaea. This observation is consistent with other surveys, focused on thermal aquifers, characterized by temperature range of 45–55 °C, and neutral or slightly alkaline pH [1,2,60]. At the phyla level, Firmicutes and Proteobacteria dominated in all sampled habitats, which is in line with other reports [3]. On the other hand, the representatives of both of these phyla can be characterized by very diverse types of metabolism. Indeed, on the lower taxonomy levels we observed much higher variability between sampled sites with dominant taxa, defined by the specificity of water chemical composition. This makes the microbial communities unique to some extent.

### 4.2. Correlation of Microbial Taxa with Elemental Composition of Water and Environmental Condition

Thermal habitats are very diverse by their origin, and include terrestrial hot springs, volcanic calderas, subterranean thermal aquifers, and oil reservoirs, etc. Temperature is considered to be the key factor determining the composition of microbial communities [61]; however, recent biogeographical screens reported that communities thriving at temperatures less than 70 °C are rather shaped by pH than temperature [1]. The chemical composition of thermal waters also plays a crucial role, defining available carbon sources, electron donors, and acceptors. In present study, three thermal habitats, while fairly similar in temperature and pH, differed significantly in the source of thermal fluid, water salinity, and chemical composition (Table 1 and Table 2; Supplementary Figure S1). Due to this fact, we observed a high level of clusterization of sampling sites on taxonomical ordination plots (Figure 4; Supplementary Figure S2). Canonical correspondence analysis revealed the determining parameter in the studied set of samples was the content of sulfur compounds. It strongly agreed with analysis of predicted metabolic properties of dominant taxa: there were several bacterial genera that could oxidize reduced sulfur compounds with oxygen as well as perform sulfur disproportionation (Supplementary Table S5). Interestingly, microbial composition in some springs has stronger correlation with several rare elements concentration, in particular, zirconium, indium, tin, and arsenic showed than temperature. Since the biological role of these elements (except for arsenic salts, which are capable to serve as the electron acceptors) is unclear, we suppose that this observation is rather linked correlations between different elements in the sampling sites.

### 4.3. Serial Filtering of Water Samples as a Strategy for Preliminary Insights on Ecological Niches and Better Resolution of Low Abundant Taxa

Filtration of water samples is the most widely used method for studying aquatic microbial ecosystems, allowing the efficient concentration of biomass from large volumes of liquid even at low cell concentrations. However, the classical methods of filtering through 0.45 or 0.22 μm filters inevitably impose a few limitations on the interpretation of the results. First, part of the water column microbial community is associated with particles of organic or inorganic matter, which inevitably lead to filter blockage. At the same time, the use of prefilters significantly distorts the composition of the microbial communities [18,19]. Second, “small microbial forms’’ often pass freely through a 0.45 µm filter, resulting in the loss of information about a significant portion of the microbial community [62].

In our work we applied a serial filtration strategy using 0.45 and 0.22 µm filters. This allowed us, on the one hand, to make reasonable assumptions about the association of microorganisms with mineral particles and, on the other hand, to detect with high confidence several lineages of candidate phyla radiation that probably would not have been identified using the standard filtering strategy.

### 4.4. Detection of Deep and Possibly Endemic Lineages of Uncultivated Microorganisms

As a result of a number of metabarcoding and metagenomic screens, the existence of a vast number of deep uncultured phylogenetic lineages significantly expanded the tree of life [63]. However, recent studies still continue to reveal new candidate taxa derived from metagenome-assembled genomes [64,65]. Despite deep shotgun sequencing followed by metagenomic binning being the method of choice for reliable phylogenetic positioning of novel uncultured candidate taxa, the 16S rRNA metabarcoding may serve as a method for surveying microbial communities for the presence of novel microbial lineages.

In our study we detected a few potentially deep phylogenetic lineages, including uncultured Firmicutes bacteria probably belonging to candidate classes TSAC18 (ASV0001, ASV0084) and D8A-2 (ASV0091), uncultured Ignavibacteriales (Bacteroidota phylum) bacterium from candidate lineage SR-FBR-L83 (ASV0024); unclassified Parcubacteria within Patescibacteria (ASV0051) and bacterium from candidate phylum Omnitrophica (ASV0054). Since these microorganisms were detected among the most abundant taxa, we suppose they might play an important role in biogeochemical cycling occurring in the hot springs. Interestingly, search of close relatives in NCBI nr/nt database showed that almost none of these uncultivated taxa had hits with more than 97% identity, which might point to possible endemism at least at lower taxonomic levels (Supplementary Table S6). That might point to potential endemism of these lineages, driven by the isolated nature of studied environments and unique combination of chemical parameters, especially in the Karmadon springs, located in the area of potential volcanism. This observation lies in line with recent work by Louca on the dispersal rate of microorganisms, who reported a much lower dispersion rate of thermophilic microorganisms, as opposed to mesophilic or human-associated taxa [66]. Moreover, the endemic taxa of thermophiles have recently been reported in several comparative studies [10,67,68].

## 5. Conclusions

Here, we report a first culture-independent (16S rRNA metabarcoding) survey of microbial communities inhabiting hot springs in the Republic of North Ossetia-Alania (North Caucasus, Russia). We sampled two deep subterranean thermal aquifers and one hydrothermal field with the temperature in the range of 43–55 °C. In total, representatives of 50 phyla were detected with Bacteria having total dominance over Archaea. The most abundant phyla were Proteobacteria, Firmicutes, and Bacteroidota which represented ~70 to 80% of the communities. In filtered water fractions, ultrasmall bacteria such as Elusimicrobiota and Patescibacteria were found. The microbial community of the terrestrial Karmadon hot springs is significantly influenced by anthropogenic factors but is dominated by moderate thermophiles. The majority of identified cultivated microorganisms in the Karmadon springs is heterotrophic, obviously due to the large amounts of allochtonic organic matter in these springs. In turn, subterranean aquifers were characterized by much larger numbers of obligate autotrophs.

Canonical correspondence analysis of correlations of environmental factors with the composition of microbial communities showed that for our dataset the content of sulfur compounds played a decisive role, which corresponded with presence of numerous bacteria, probably metabolized sulfur compounds (respiration with oxygen as acceptor and disproportionation); however, the specific content of other elements also was significant.

A few deep lineages of uncultivated microorganisms were detected among the most abundant taxa: uncultured Firmicutes (TSAC18 and D8A-2 lineages), uncultured Bacteroidota (SR-FBR-L83 group within Ignavibacteriales), and unclassified Patescibacteria (candidate class Parcubacteria). This endemic part of the communities seems to play an important role in metabolic circuits of these microbial consortia.

## Figures and Tables

**Table 1:** Physicochemical characteristics of North Ossetian geothermal habitats.

Location	Latitude Longitude	Altitude	Depth of the Well	T, °C	pH	Eh	Enriched Elements
**Biragzang**	42.9957 N, 44.2290 E	685 m	2370 m	48	8.8	−20–0	Sb, V, Al, Mo, Ga, Cd
**Karmadon**	42.7547 N, 44.4811 E	2330 m	NA	52–55	6.1	−20–0	K, Si, B, As, Fe, Li, Rb, Mn, Be, Ba
**Ursdon**	43.0626 N, 44.0490 E	643 m	1530 m	45–47	7.0	−370–140 *	S, Mg, Ca Sn, Zr, In

* Extremely reduced pH values were detected in hydrosulfide-rich puddle formed as a result of thermal water pipe seepage near the Ursdon well outlet.

**Table 2:** Chemical composition of North Ossetian thermal water *.

	Concentration of Major Elements, µg/L								
	**B**	**Na**	**Mg**	**Al**	**Si**	**S**	**K**	**Ca**	**V**
Biragzang	2121.0	269,292.0	46.7	52.8	14,676.0	17,184.0	1458.0	904.0	0.7
Karmadon #4135	62,696.0	1,975,454.0	44,306.0	<LOD	27,751.0	40,247.0	295,956.0	356,082.0	<LOD
Karmadon #4138	72,680.3	2,126,284.0	47,948.0	<LOD	29,430.0	43,329.0	318,907.0	389,111.0	<LOD
Ursdon	7967.0	1,571,141.0	171,799.0	<LOD	14,327.0	867,285.0	62,203.0	651,088.0	<LOD
LOD	2.0	3.0	4.0	1.0	5.0	16.0	74.0	110.0	0.2
	**Mn**	**Fe**	**Zn**	**Ge**	**As**	**Br**	**Sr**	**Ba**	**Pb**
Biragzang	0.4	<LOD	1.9	4.8	330.0	181.0	66.5	31.3	<LOD
Karmadon #4135	761.0	3254.0	176.0	20.2	442.0	7120.0	10,243.0	345.0	0.6
Karmadon #4138	784.0	3265.0	243.0	22.2	540.0	7805.0	11,102.0	364.0	<LOD
Ursdon	6.2	<LOD	398	3.3	<LOD	7942.0	15,624.0	4.9	<LOD
LOD	0.2	7.0	0.6	0.1	0.1	8.0	0.6	0.4	0.3
	**Concentration of Rare Elements** **, ng/L**								
	**Li**	**Be**	**Rb**	**Y**	**Zr**	**Mo**	**Cd**	**In**	**Sn**
Biragzang	41,785.0	<LOD	1937.0	<LOD	<LOD	15,677.0	53.2	<LOD	<LOD
Karmadon #4135	11,831,981.0	2130.0	2,120,957.0	1079.0	<LOD	485.0	<LOD	<LOD	<LOD
Karmadon #4138	12,457,781.0	3033.7	2,235,407.0	905.0	<LOD	569.0	<LOD	<LOD	<LOD
Ursdon	1,164,150.0	<LOD	68,102	<LOD	223.0	<LOD	<LOD	189.0	1025.0
LOD	80.0	6.0	10.0	3.0	6.0	30.0	6.0	3.0	29.0
	**Sb**	**Cs**	**La**	**Ce**	**Pr**	**Nd**	**Sm**	**Eu**	**Gd**
Biragzang	209.0	158.0	<LOD	<LOD	<LOD	<LOD	<LOD	<LOD	<LOD
Karmadon #4135	<LOD	2,049,997.0	160.0	171.0	22.3	156.0	26.4	21.5	39.8
Karmadon #4138	<LOD	2,166,997.0	142.0	135.0	14.5	93.1	29.0	<LOD	41.3
Ursdon	<LOD	4405.0	116	38.2	<LOD	37.7	<LOD	<LOD	<LOD
LOD	10.0	6.0	33.0	3.0	6.0	7.0	0.5	0.5	0.6
	**Dy**	**Ho**	**Er**	**Yb**	**W**	**Tl**	**Th**	**U**	
Biragzang	41,785.0	<LOD	1937.0	<LOD	<LOD	15,677.0	53.2	<LOD	
Karmadon #4135	11,831,981.0	2130.0	2,120,957.0	1079.0	<LOD	485.0	<LOD	<LOD	
Karmadon #4138	12,457,781.0	3033.7	2,235,407.0	905.0	<LOD	569.0	<LOD	<LOD	
Ursdon	1,164,150.0	<LOD	68,102	<LOD	223.0	<LOD	<LOD	189.0	
LOD	0.5	0.6	0.7	3.0	6.0	4.0	0.8	5.0	

LOD—limit of detection. * Were not detected: Sc, Ti, Cr, Co, Ni, Cu, Se, Hg, Nb, Ru, Rh, Pd, Ag, Te, Tb, Tm, Lu, Hf, Ta, Re, Os, Ir, Pt, Au, Bi.

## Data Availability

Description of the project and the metadata of sampling sites were submitted to NCBI under the Bioproject PRJNA778948. Shotgun metagenomic reads were submitted to NCBI SRA archive under run accession number SRR16889595-SRR16889613.

## Supplementary Materials

The following are available online at https://www.mdpi.com/article/10.3390/biology10121352/s1, Supplementary.pdf—Supplementary Tables and Figures [34,37,66,69,70,71,72,73,74,75,76,77,78,79,80,81,82,83,84,85,86,87,88,89].

## References

[1] Power J.F., Carere C.R., Lee C.K., Wakerley G.L.J., Evans D.W., Button M., White D., Climo M.D., Hinze A.M., Morgan X.C. (2018). Microbial Biogeography of 925 Geothermal Springs in New Zealand. Nat. Commun..

[2] Chan C.S., Chan K.-G., Ee R., Hong K.-W., Urbieta M.S., Donati E.R., Shamsir M.S., Goh K.M. (2017). Effects of Physiochemical Factors on Prokaryotic Biodiversity in Malaysian Circumneutral Hot Springs. Front. Microbiol..

[3] Massello F.L., Chan C.S., Chan K.-G., Goh K.M., Donati E., Urbieta M.S. (2020). Meta-Analysis of Microbial Communities in Hot Springs: Recurrent Taxa and Complex Shaping Factors beyond PH and Temperature. Microorganisms.

[4] Purcell D., Sompong U., Yim L.C., Barraclough T.G., Peerapornpisal Y., Pointing S.B. (2007). The Effects of Temperature, PH and Sulphide on the Community Structure of Hyperthermophilic Streamers in Hot Springs of Northern Thailand: Hyperthermophilic Diversity and Abiotic Variables. FEMS Microbiol. Ecol..

[5] Fontaneto D., Hortal J. (2012). Microbial Biogeography: Is Everything Small Everywhere?. Microbial Ecological Theory: Current Perspectives.

[6] Jones D.S., Schaperdoth I., Macalady J.L. (2016). Biogeography of Sulfur-Oxidizing Acidithiobacillus Populations in Extremely Acidic Cave Biofilms. ISME J..

[7] Dobbs F., Selph K. (1997). Thermophilic Bacterial Activity in a Deep-Sea Sediment from the Pacific Ocean. Aquat. Microb. Ecol..

[8] Price M.T., Fullerton H., Moyer C.L. (2015). Biogeography and Evolution of Thermococcus Isolates from Hydrothermal Vent Systems of the Pacific. Front. Microbiol..

[9] Beblo K., Rabbow E., Rachel R., Huber H., Rettberg P. (2009). Tolerance of Thermophilic and Hyperthermophilic Microorganisms to Desiccation. Extremophiles.

[10] Podar P.T., Yang Z., Björnsdóttir S.H., Podar M. (2020). Comparative Analysis of Microbial Diversity Across Temperature Gradients in Hot Springs From Yellowstone and Iceland. Front. Microbiol..

[11] Zaalishvili V.B., Nevskaya N.I., Nevskii L.N., Shempelev A.G. (2015). Geophysical Fields above Volcanic Edifices in the North Caucasus. J. Volcanol. Seism..

[12] Mintsaev M.S., Machigova F., Khadasheva Z., Cherkasov S., Churikova T. (2016). Mineral Resources of the Geothermal Sources of the North Caucasus. Int. J. Environ. Sci. Educ..

[13] Khalilova E.A., Nuratinov R.A., Kotenko S.C., Islammagomedova E.A. (2014). Hydrocarbon-Oxidizing Microorganisms of Hot Springs and Their Significance in the Assessment of the Biodiversity of Microbial Communities. Arid. Ecosyst..

[14] Chernousova E.Y., Akimov V.N., Gridneva E.V., Dubinina V.A., Grabovich M.Y. (2010). Erratum to: “Biodiversity and Monitoring of Colorless Filamentous Bacteria in Sulfide Aquatic Systems of North Caucasus Region”. Microbiology.

[15] Chernousova E.Y., Akimov V.N., Gridneva E.V., Dubinina G.A., Grabovich M.Y. (2008). Phylogenetic in Situ/Ex Situ Analysis of a Sulfur Mat Microbial Community from a Thermal Sulfide Spring in the North Caucasus. Microbiology.

[16] Kochetkova T., Podosokorskaya O., Elcheninov A., Kublanov I. Diversity of Thermophilic Prokaryotes Inhabiting Russian Natural Hot Springs; 2022.

[17] Zaalishvili V.B., Melkov D.A., Burdzieva O.G. (2015). Possibilities of Geothermal Energy Use on the North Caucasus (a View on a Problem from Azores Example). Ecol. Environ. Conserv..

[18] Torres-Beltrán M., Mueller A., Scofield M., Pachiadaki M.G., Taylor C., Tyshchenko K., Michiels C., Lam P., Ulloa O., Jürgens K. (2019). Sampling and Processing Methods Impact Microbial Community Structure and Potential Activity in a Seasonally Anoxic Fjord: Saanich Inlet, British Columbia. Front. Mar. Sci..

[19] Azam F., Malfatti F. (2007). Microbial Structuring of Marine Ecosystems. Nat. Rev. Microbiol..

[20] Gohl D., Gohl D.M., MacLean A., Hauge A., Becker A., Walek D., Beckman K.B. (2016). An Optimized Protocol for High-Throughput Amplicon-Based Microbiome Profiling. Protoc. Exch..

[21] Hugerth L.W., Wefer H.A., Lundin S., Jakobsson H.E., Lindberg M., Rodin S., Engstrand L., Andersson A.F. (2014). DegePrime, a Program for Degenerate Primer Design for Broad-Taxonomic-Range PCR in Microbial Ecology Studies. Appl. Environ. Microbiol..

[22] Merkel A.Yu., Tarnovetskii I.Yu., Podosokorskaya O.A., Toshchakov S.V. (2019). Analysis of 16S RRNA Primer Systems for Profiling of Thermophilic Microbial Communities. Microbiology.

[23] Kochetkova T.V., Toshchakov S.V., Zayulina K.S., Elcheninov A.G., Zavarzina D.G., Lavrushin V.Yu., Bonch-Osmolovskaya E.A., Kublanov I.V. (2020). Hot in Cold: Microbial Life in the Hottest Springs in Permafrost. Microorganisms.

[24] Renaud G., Stenzel U., Maricic T., Wiebe V., Kelso J. (2015). DeML: Robust Demultiplexing of Illumina Sequences Using a Likelihood-Based Approach. Bioinformatics.

[25] Callahan B.J., Mcmurdie P.J., Rosen M.J., Han A.W., Johnson A.J.A., Holmes S.P. (2016). DADA2: High-resolution sample inference from Illumina amplicon data. Nat. Methods.

[26] Quast C., Pruesse E., Yilmaz P., Gerken J., Schweer T., Yarza P., Peplies J., Glöckner F.O. (2012). The SILVA Ribosomal RNA Gene Database Project: Improved Data Processing and Web-Based Tools. Nucleic Acids Res..

[27] McMurdie P.J., Holmes S. (2013). Phyloseq: An R Package for Reproducible Interactive Analysis and Graphics of Microbiome Census Data. PLoS ONE.

[28] Davis N.M., Proctor D.M., Holmes S.P., Relman D.A., Callahan B.J. (2018). Simple Statistical Identification and Removal of Contaminant Sequences in Marker-Gene and Metagenomics Data. Microbiome.

[29] Vegan: Community Ecology Package. Ordination Methods, Diversity Analysis and Other Functions for Community and Vegetation Ecologists. https://www.worldagroforestry.org/publication/vegan-community-ecology-package-ordination-methods-diversity-analysis-and-other.

[30] Lahti L., Shetty S., Blake T., Salojarvi J. Microbiome; Version 1.0.2; Bioconductor: 2017. https://bioconductor.statistik.tu-dortmund.de/packages/3.6/bioc/html/microbiome.html.

[31] Hsieh T.C., Ma K.H., Chao A. (2016). INEXT: An R Package for Rarefaction and Extrapolation of Species Diversity (H Ill Numbers). Methods Ecol. Evol..

[32] Dzhgamadze A.K., Gogichev R.R., Dzeranov B.V. Hydrochemical Characteristics of the Biragzang Groundwater Area; 2019.

[33] Lavrushin V.Y., Kuleshov V.N., Kikvadze O.E. (2006). Travertines of the Northern Caucasus. Lithol. Miner. Resour..

[34] Dzgoev U.S. (1961). The Karmadon Resort.

[35] Boden R., Hutt L.P., Rae A.W. (2017). Reclassification of Thiobacillus Aquaesulis (Wood & Kelly, 1995) as Annwoodia Aquaesulis Gen. Nov., Comb. Nov., Transfer of Thiobacillus (Beijerinck, 1904) from the Hydrogenophilales to the Nitrosomonadales, Proposal of Hydrogenophilalia Class. Nov. within the ‘Proteobacteria’, and Four New Families within the Orders Nitrosomonadales and Rhodocyclales. Int. J. Syst. Evol. Microbiol..

[36] Bagdigian R.M., Myerson A.S. (1986). The Adsorption OfThiobacillus Ferrooxidans on Coal Surfaces. Biotechnol. Bioeng..

[37] Myerson A.S., Kline P. (1983). The Adsorption OfThiobacillus Ferrooxidans on Solid Particles. Biotechnol. Bioeng..

[38] Mori K., Suzuki K. (2008). Thiofaba Tepidiphila Gen. Nov., Sp. Nov., a Novel Obligately Chemolithoautotrophic, Sulfur-Oxidizing Bacterium of the Gammaproteobacteria Isolated from a Hot Spring. Int. J. Syst. Evol. Microbiol..

[39] Yoshida N., Takahashi N., Hiraishi A. (2005). Phylogenetic Characterization of a Polychlorinated-Dioxin- Dechlorinating Microbial Community by Use of Microcosm Studies. Appl. Environ. Microbiol..

[40] Zeglin L.H., Wang B., Waythomas C., Rainey F., Talbot S.L. (2016). Organic Matter Quantity and Source Affects Microbial Community Structure and Function Following Volcanic Eruption on Kasatochi Island, Alaska: Microbial Structure and Function after Volcanic Eruption. Environ. Microbiol..

[41] Sokolova T.G., Kostrikina N.A., Chernyh N.A., Tourova T.P., Kolganova T.V., Bonch-Osmolovskaya E.A. (2002). Carboxydocella Thermautotrophica Gen. Nov., Sp. Nov., a Novel Anaerobic, CO-Utilizing Thermophile from a Kamchatkan Hot Spring. Int. J. Syst. Evol. Microbiol..

[42] Toshchakov S.V., Lebedinsky A.V., Sokolova T.G., Zavarzina D.G., Korzhenkov A.A., Teplyuk A.V., Chistyakova N.I., Rusakov V.S., Bonch-Osmolovskaya E.A., Kublanov I.V. (2018). Genomic Insights Into Energy Metabolism of Carboxydocella Thermautotrophica Coupling Hydrogenogenic CO Oxidation With the Reduction of Fe(III) Minerals. Front. Microbiol..

[43] Lee J., Koo T., Yulisa A., Hwang S. (2019). Magnetite as an Enhancer in Methanogenic Degradation of Volatile Fatty Acids under Ammonia-Stressed Condition. J. Environ. Manag..

[44] Korzhenkov A., Teplyuk A.V., Lebedinsky A.V., Khvashchevskaya A.A., Kopylova Y.G., Arakchaa K.D., Golyshin P., Lunev E., Golyshina O.V., Kublanov I.V. (2018). Members of the Uncultured Taxon OP1 (“Acetothermia”) Predominate in the Microbial Community of an Alkaline Hot Spring at East-Tuvinian Upland. Microbiology.

[45] Schuler C.G., Havig J.R., Hamilton T.L. (2017). Hot Spring Microbial Community Composition, Morphology, and Carbon Fixation: Implications for Interpreting the Ancient Rock Record. Front. Earth Sci..

[46] Hidalgo K.J., Teramoto E.H., Soriano A.U., Valoni E., Baessa M.P., Richnow H.H., Vogt C., Chang H.K., Oliveira V.M. (2020). Taxonomic and Functional Diversity of the Microbiome in a Jet Fuel Contaminated Site as Revealed by Combined Application of in Situ Microcosms with Metagenomic Analysis. Sci. Total Environ..

[47] Méheust R., Castelle C.J., Matheus Carnevali P.B., Farag I.F., He C., Chen L.-X., Amano Y., Hug L.A., Banfield J.F. (2020). Groundwater Elusimicrobia Are Metabolically Diverse Compared to Gut Microbiome Elusimicrobia and Some Have a Novel Nitrogenase Paralog. ISME J..

[48] Kotlyakov V.M., Rototaeva O.V., Nosenko G.A., Chernov R.A. (2015). Ten years after the Karmadon catastrophe, North Ossetia: On the causes of event and the glacier recovery processes. Izv. Ross. Akad. Nauk. Seriya Geogr..

[49] Wackett L.P., Dodge A.G., Ellis L.B.M. (2004). Microbial Genomics and the Periodic Table. Appl. Environ. Microbiol..

[50] Xiu W., Lloyd J., Guo H., Dai W., Nixon S., Bassil N.M., Ren C., Zhang C., Ke T., Polya D. (2020). Linking microbial community composition to hydrogeochemistry in the western Hetao Basin: Potential importance of ammonium as an electron donor during arsenic mobilization. Environ. Int..

[51] Podosokorskaya O.A., Kochetkova T.V., Novikov A.A., Toshchakov S.V., Elcheninov A.G., Kublanov I.V. (2020). *Tenuifilum thalassicum gen. nov., sp. nov*., a novel moderate thermophilic anaerobic bacterium from a Kunashir Island shallow hot spring representing a new family Tenuifilaceae fam. nov. in the class Bacteroidia. Syst. Appl. Microbiol..

[52] Alkhasov A.B., Alkhasova D.A. (2014). Up-to-Date State and Prospects for the Development of Geothermal Resources of the North Caucasus Region. Therm. Eng..

[53] Ershov A.V., Brunet M.-F., Nikishin A.M., Bolotov S.N., Nazarevich B.P., Korotaev M.V. (2003). Northern Caucasus Basin: Thermal History and Synthesis of Subsidence Models. Sediment. Geol..

[54] Aburto-Medina A., Shahsavari E., Cohen M., Mantri N., Ball A.S. (2020). Analysis of the Microbiome (Bathing Biome) in Geothermal Waters from an Australian Balneotherapy Centre. Water.

[55] Hirayama H., Takai K., Inagaki F., Nealson K.H., Horikoshi K. (2005). *Thiobacter subterraneus gen. nov., sp. nov*., an obligately chemolithoautotrophic, thermophilic, sulfur-oxidizing bacterium from a subsurface hot aquifer. Int. J. Syst. Evol. Microbiol..

[56] França L., Rainey F.A., Nobre M.F., da Costa M.S. (2006). *Tepidicella xavieri gen. nov., sp. nov*., a betaproteobacterium isolated from a hot spring runoff. Int. J. Syst. Evol. Microbiol..

[57] Albuquerque L., Egas C. (2021). Tepidimonas. Bergey’s Manual of Systematics of Archaea and Bacteria.

[58] Frank Y.A., Kadnikov V.V., Gavrilov S.N., Banks D., Gerasimchuk A.L., Podosokorskaya O.A., Merkel A.Y., Chernyh N.A., Mardanov A.V., Ravin N.V. (2016). Stable and Variable Parts of Microbial Community in Siberian Deep Subsurface Thermal Aquifer System Revealed in a Long-Term Monitoring Study. Front. Microbiol..

[59] Kalwasińska A., Krawiec A., Deja-Sikora E., Gołębiewski M., Kosobucki P., Swiontek Brzezinska M., Walczak M. (2020). Microbial Diversity in Deep-Subsurface Hot Brines of Northwest Poland: From Community Structure to Isolate Characteristics. Appl. Environ. Microbiol..

[60] Stout L.M., Blake R.E., Greenwood J.P., Martini A.M., Rose E.C. (2009). Microbial Diversity of Boron-Rich Volcanic Hot Springs of St. Lucia, Lesser Antilles. FEMS Microbiol. Ecol..

[61] Sharp C.E., Smirnova A.V., Graham J.M., Stott M.B., Khadka R., Moore T.R., Grasby S.E., Strack M., Dunfield P.F. (2014). Distribution and Diversity of *V Errucomicrobia* Methanotrophs in Geothermal and Acidic Environments: Diversity of Verrucomicrobial Methanotrophs. Environ. Microbiol..

[62] Vigneron A., Cruaud P., Langlois V., Lovejoy C., Culley A.I., Vincent W.F. (2020). Ultra-small and abundant: Candidate phyla radiation bacteria are potential catalysts of carbon transformation in a thermokarst lake ecosystem. Limnol. Oceanogr. Lett..

[63] Castelle C.J., Wrighton K.C., Thomas B.C., Hug L.A., Brown C.T., Wilkins M.J., Frischkorn K.R., Tringe S.G., Singh A., Markillie L.M. (2015). Genomic expansion of domain archaea highlights roles for organisms from new phyla in anaerobic carbon cycling. Curr. Biol..

[64] Vavourakis C.D., Andrei A.-S., Mehrshad M., Ghai R., Sorokin D.Y., Muyzer G. (2018). A Metagenomics Roadmap to the Uncultured Genome Diversity in Hypersaline Soda Lake Sediments. Microbiome.

[65] Zhu J., Tian L., Chen P., Han M., Song L., Tong X., Sun X., Yang F., Lin Z., Liu X. (2021). Over 50,000 Metagenomically Assembled Draft Genomes for the Human Oral Microbiome Reveal New Taxa. Genom. Proteom. Bioinform..

[66] Louca S. (2021). The Rates of Global Bacterial and Archaeal Dispersal. ISME J..

[67] Papke R.T., Ramsing N.B., Bateson M.M., Ward D.M. (2003). Geographical Isolation in Hot Spring Cyanobacteria: Geographical Isolation in Hot Spring Cyanobacteria. Environ. Microbiol..

[68] Takacs-Vesbach C., Mitchell K., Jackson-Weaver O., Reysenbach A.-L. (2008). Volcanic Calderas Delineate Biogeographic Provinces among Yellowstone Thermophiles. Environ. Microbiol..

[69] Killham K., Prosser J.I., Dworkin M., Falkow S., Rosenberg E., Schleifer K.-H., Stackebrandt E. (2006). The prokaryotes. Soil Microbiology, Ecology and Biochemistry.

[70] Cunha S., Tiago I., Pires A.L., Da Costa M.S., Veríssimo A. (2006). Dokdonella fugitiva sp. nov., a Gammaproteobacterium isolated from potting soil. Syst. Appl. Microbiol..

[71] Grimont P.A.D., Grimont F. (2015). Klebsiella. Bergey’s Manual of Systematics of Archaea and Bacteria.

[72] Palleroni N.J. (2015). Pseudomonas. Bergey’s Manual of Systematics of Archaea and Bacteria.

[73] Albuquerque L., Santos J., Travassos P., Nobre M.F., Rainey F.A., Wait R., Empadinhas N., Silva M.T., Da Costa M.S. (2002). Albidovulum inexpectatum gen. nov., sp. nov., a Nonphotosynthetic and slightly thermophilic bacterium from a marine hot spring that is very closely related to members of the photosynthetic genus Rhodovulum. Appl. Environ. Microbiol..

[74] Podosokorskaya O.A., Kadnikov V.V., Gavrilov S.N., Mardanov A.V., Merkel A.Y., Karnachuk O.V., Ravin N.V., Bonch-Osmolovskaya E.A., Kublanov I.V. (2013). Characterization of Melioribacter roseus gen. nov., sp. nov., a novel facultatively anaerobic thermophilic cellulolytic bacterium from the class Ignavibacteria, and a proposal of a novel bacterial phylum Ignavibacteriae. Environ. Microbiol..

[75] Proença D.N., Whitman W.B., Varghese N., Shapiro N., Woyke T., Kyrpides N.C., Morais P.V. (2018). Arboriscoccus pini gen. nov., sp. nov., an endophyte from a pine tree of the class Alphaproteobacteria, emended description of Geminicoccus roseus, and proposal of Geminicoccaceae fam. nov. Syst. Appl. Microbiol..

[76] Albuquerque L., Rainey F.A., Nobre M.F., da Costa M.S. (2011). Schleiferia thermophila gen. nov., sp. nov., a slightly thermophilic bacterium of the phylum “bacteroidetes” and the proposal of schleiferiaceae fam. nov. Int. J. Syst. Evol. Microbiol..

[77] Iino T., Ohkuma M., Kamagata Y., Amachi S. (2016). Iodidimonas muriae gen. Nov., sp. nov., an aerobic iodide-oxidizing bacterium isolated from brine of a natural gas and iodine recovery facility, and proposals of Iodidimonadaceae fam. nov., Iodidimonadales ord. nov., Emcibacteraceae fam. nov. and Emcibact. Int. J. Syst. Evol. Microbiol..

[78] Albuquerque L., Polónia A.R.M., Barroso C., Froufe H.J.C., Lage O., Lobo-Da-Cunha A., Egas C., da Costa M.S. (2018). Raineya orbicola gen. nov., sp. nov. a slightly thermophilic bacterium of the phylum bacteroidetes and the description of raineyaceae fam. nov. Int. J. Syst. Evol. Microbiol..

[79] Chen Z., Lei X., Lai Q., Li Y., Zhang B., Zhang J., Zhang H., Yang L., Zheng W., Tian Y. (2014). Phaeodactylibacter xiamenensis gen. nov., sp. nov., a member of the family Saprospiraceae isolated from the marine alga Phaeodactylum tricornutum. Int. J. Syst. Evol. Microbiol..

[80] Bovre K., Henriksen S.D. (1967). A new Moraxella species, Moraxella osloensis, and a revised description of Moraxella nonliquefaciens. Int. J. Syst. Bacteriol..

[81] Slobodkina G.B., Baslerov R.V., Novikov A.A., Bonch-Osmolovskaya E.A., Slobodkin A.I. (2017). Thermodesulfitimonas autotrophica gen. Nov., sp. Nov., a thermophilic, obligate sulfite-reducing bacterium isolated from a terrestrial hot spring. Int. J. Syst. Evol. Microbiol..

[82] Busse H.-J. (2015). Micrococcus. Bergey’s Manual of Systematics of Archaea and Bacteria.

[83] Loginova L.G., Egorova L.A., Golovacheva R.S., Seregina L.M. (1984). Thermus ruber sp. nov., nom. rev. Int. J. Syst. Bacteriol..

[84] Schumacher W., Kroneck P.M.H., Pfennig N. (1992). Comparative systematic study on “Spirillum” 5175, Campylobacter and Wolinella species—Description of “Spirillum” 5175 as Sulfurospirillum deleyianum gen. nov., spec. nov. Arch. Microbiol..

[85] Umezawa K., Kojima H., Kato Y., Fukui M. (2021). Dissulfurispira thermophila gen. nov., sp. nov., a thermophilic chemolithoautotroph growing by sulfur disproportionation, and proposal of novel taxa in the phylum Nitrospirota to reclassify the genus Thermodesulfovibrio. Syst. Appl. Microbiol..

[86] Slobodkina G.B., Kolganova T.V., Kopitsyn D.S., Viryasov M.B., Bonch-Osmolovskaya E.A., Slobodkin A.I. (2016). Dissulfurirhabdus thermomarina gen. nov., sp. nov., a thermophilic, autotrophic, sulfite-reducing and disproportionating deltaproteobacterium isolated from a shallow-sea hydrothermal vent. Int. J. Syst. Evol. Microbiol..

[87] Stieglmeier M., Klingl A., Alves R.J.E., Rittmann S.K.M.R., Melcher M., Leisch N., Schleper C. (2014). Nitrososphaera viennensis gen. nov., sp. nov., an aerobic and mesophilic, ammonia-oxidizing archaeon from soil and a member of the archaeal phylum Thaumarchaeota. Int. J. Syst. Evol. Microbiol..

[88] Koelschbach J.S., Mouttaki H., Pickl C., Heipieper H.J., Rache R., Lawson P.A., Meckenstock R.U. (2017). Rectinema cohabitans gen. nov., sp. nov., a rod-shaped spirochaete isolated from an anaerobic naphthalene-degrading enrichment culture. Int. J. Syst. Evol. Microbiol..

[89] Pohlschroeder M., Leschine S.B., Canale-Parola E. (1994). Spirochaeta caldaria sp. nov., a thermophilic bacterium that enhances cellulose degradation by Clostridium thermocellum. Arch. Microbiol..

