# Supplementary material for: Culture-Independent Survey of Thermophilic Microbial Communities of the North Caucasus
Source: Biology (Basel). 2021 Dec 20;10(12):1352. doi: 10.3390/biology10121352 (PMC8698779; doi:10.3390/biology10121352)

SUPPLEMENTARY MATERIALS

First culture-independent survey of thermophilic microbial communities of North Caucasus.

Toshchakov SV[1],*, Izotova AO[1], Vinogradova EN[1], Kachmazov GS[2], Tuaeva AY[3], Abaev VT[2], Evteeva MA[1], Gunitseva NM[1], Korzhenkov AA[1], Elcheninov AG[4], Patrushev MV[1], Kublanov IV[4]

[1] Kurchatov Center for Genome Research, National Research Center "Kurchatov Institute", Ac. Kurchatov square, 1, 123098 Moscow, Russia; nrcki@nrcki.ru

[2] North Ossetian State University named after K.L. Khetagurov, Vatutina str., 44-46, 362025, Vladikavkaz, Republic North Ossetia-Alania, Russia; nosu@nosu.ru

[3] Winogradsky Institute of Microbiology, Research Center of Biotechnology RAS, 60-let Oktyzbrya Av., 7/2, 119071, Moscow, Russia; inmi@inmi.ru

[4] National Research Center Kurchatov Institute-GOSNIIGENETIKA, 1st Dorozhny Pr., 1, 117545, Moscow, Russia; vkpm@genetika.ru

* Correspondence: stepan.toshchakov@gmail.com; Tel.: +7-911-481-1809

SUPPLEMENTARY TABLES

Supplementary Table S1. Tracking of read number of V4 16S rRNA dataset, processed with DADA2 pipeline

Sample.names	input	filtered	denoisedF	denoisedR	merged	nonchim
**alania.4128.filter.0.22.V4.repl1**	14774	14305	14056	14175	13555	13530
**alania.4128.filter.0.22.V4.repl2**	17167	16541	16338	16390	15745	15725
**alania.4128.filter.0.45.V4.repl1**	28240	27700	27384	27474	25649	23974
**alania.4128.filter.0.45.V4.repl2**	26680	26032	25745	25853	24062	22262
**alania.4128.tube.V4.repl1**	26652	26128	25712	25860	24196	24096
**alania.4128.tube.V4.repl2**	27907	27369	26936	27064	25195	24954
**alania.4129A.tube.V4.repl1**	16767	16337	16214	16241	15955	15940
**alania.4129A.tube.V4.repl2**	17134	16691	16538	16583	16281	16253
**alania.4135.filter.0.22.V4.repl1**	15369	14959	14101	14440	12461	12391
**alania.4135.filter.0.22.V4.repl2**	15075	14746	14031	14232	12673	12599
**alania.4135.filter.0.45.V4.repl1**	23244	22608	21280	21976	18951	18877
**alania.4135.filter.0.45.V4.repl2**	18791	18342	17200	17650	15260	15201
**alania.4135.tube.V4.repl1**	22057	21669	21304	21435	19103	17968
**alania.4135.tube.V4.repl2**	31948	31236	30676	30937	27913	26029
**alania.4138.filter.0.22.V4.repl1**	13102	12823	12540	12608	12055	11975
**alania.4138.filter.0.22.V4.repl2**	16136	15773	15425	15587	14749	14728
**alania.4138.filter.0.45.V4.repl1**	21931	21593	21262	21384	20410	20088
**alania.4138.filter.0.45.V4.repl2**	25034	24702	24407	24493	23361	22891
**alania.4139.tube.V4.repl1**	23777	23287	23040	23079	21911	21412
**alania.4139.tube.V4.repl2**	31222	30727	30468	30529	29309	28320
**alania.4146.filter.0.22.V4.repl1**	26043	25230	24328	24691	22273	20538
**alania.4146.filter.0.22.V4.repl2**	25345	24703	23794	24175	21819	19897
**alania.4146.filter.0.45.V4.repl1**	23983	23557	22279	22940	19851	17321
**alania.4146.filter.0.45.V4.repl2**	15418	15001	13988	14500	12572	11371
**alania.4148.tube.V4.repl1**	20186	19857	18447	19136	14617	14454
**alania.4148.tube.V4.repl2**	18646	18267	16846	17654	13409	13316
**Total**	**562628**	**550183**	**534339**	**541086**	**493335**	**476110**

Supplementary Table S2. Alpha diversity metrics of analyzed communities

Sample ID	observed	chao1	diversity shannon	evenness pielou	evenness simpson	dominance simpson
**alania.4128.filter.0.22**	90	90	2.94	0.65	0.11	0.1
**alania.4128.filter.0.45**	76	76	2.47	0.57	0.07	0.19
**alania.4128.tube**	185	185.9	3.85	0.74	0.13	0.04
**alania.4129A.tube**	64	64	2.61	0.63	0.13	0.12
**alania.4135.filter.0.22**	269	277	4.03	0.72	0.09	0.04
**alania.4135.filter.0.45**	233	245.25	3.41	0.63	0.05	0.09
**alania.4135.tube**	159	161.67	3.59	0.71	0.12	0.05
**alania.4138.filter.0.22**	143	151	3.01	0.61	0.05	0.13
**alania.4138.filter.0.45**	103	105	2.9	0.63	0.09	0.11
**alania.4138.tube**	82	83.13	2.79	0.63	0.12	0.11
**alania.4146.filter.0.22**	198	216	3.54	0.67	0.08	0.07
**alania.4146.filter.0.45**	132	135.6	2.11	0.43	0.03	0.28
**alania.4148.tube**	322	322.64	4.56	0.79	0.1	0.03

Supplementary Table S3. Results of BLAST search of sequences, similar to ASV0001 in publicly available 16S and metagenomic databases

Database	Scientific Name	Query Cover	E value	Per. ident	Accession	Isolation source
NCBI 16S rRNA sequences (Bacteria and Archaea)	Thermanaeromonas burensis	99%	1.00E-80	88.19	NR_147776.1	subterranean Callovo-Oxfordian clay formation, France
	Moorella humiferrea	99%	2.00E-78	87.7	NR_108634.1	terrestrial hydrothermal spring, Kamchatka, Russia
	Thermanaeromonas toyohensis ToBE	99%	2.00E-78	87.6	NR_024777.1	geothermal aquifer in the Toyoha Mines, Japan
	Carboxydothermus hydrogenoformans	99%	6.00E-78	87.75	NR_074395.1	hot swamp of Kunashir Island, Russia
	Carboxydothermus siderophilus	99%	6.00E-78	87.75	NR_044272.1	terrestrial hydrothermal spring, Kamchatka, Russia
NCBI nucleotide collection (nr/nt)	Uncultured bacterium clone OTU48348_AL201_7080163 16S ribosomal RNA gene	100%	2.00E-110	96.02	KP930849.1	soil or post-volcanic pyroclastic surface, Alaska, USA
	Uncultured bacterium clone kab160 16S ribosomal RNA gene	100%	9.00E-99	93.23	FJ936877.1	volcano mud taken at Avachinsky volcano, Kamtchatka, Russia
	Uncultured Firmicutes bacterium clone 7150D1B63 16S ribosomal RNA gene	100%	9.00E-94	92.06	EF562164.1	deep-granitic-fracture water from Henderson Molybdenum Mine, Colorado, USA
	Uncultured Firmicutes bacterium clone 7150D1B38 16S ribosomal RNA gene	100%	4.00E-92	91.67	EF562141.1	deep-granitic-fracture water from Henderson Molybdenum Mine, Colorado, USA
	Uncultured bacterium clone BE325FW032701CTS_hole1-20 16S ribosomal RNA gene	100%	4.00E-92	91.63	DQ088766.1	fracture-derived groundwater in a deep gold mine of South Africa
JGI 16S rRNA public assembled metagenomes	Dewar_combined assembly	100%	2.00E-108	95	Ga0212093_12484561	hot spring sediment from Dewar Creek, British Columbia, Canada
	Hot spring sediment from British Columbia, Canada, Dewar Creek DC16 2012	100%	2.00E-108	95	Ga0209479_10652191	hot spring sediment from Dewar Creek, British Columbia, Canada
	Hot spring sediment from British Columbia, Canada, Dewar Creek DC16 2012	81%	4.00E-84	95	Ga0073933_11454081	hot spring sediment from Dewar Creek, British Columbia, Canada

Supplementary Table S4. Results of fitting of environmental variables in CCA ordination performed by *envfit* function of *vegan* package

EIGENVALUES

CCA1	CCA2	CCA3	CCA4	CCA5
0.9457885	0.8326652	0.7854286	0.6721891	0.5573745

Permutation: free
Number of permutations: 999

factor	CCA1	CCA2	r^2	Pr(>r)
**S**	-0.996	-0.996	0.997	0.001
**Zr**	-0.992	-0.992	0.996	0.002
**In**	-0.992	-0.992	0.996	0.002
**Sn**	-0.992	-0.992	0.996	0.002
**As**	0.855	0.855	0.990	0.001
**Mg**	-0.981	-0.981	0.988	0.001
**Zn**	-0.837	-0.837	0.972	0.001
**T, °C**	0.592	0.592	0.965	0.001
**Be**	0.359	0.359	0.955	0.001
**Ca**	-0.825	-0.825	0.954	0.001
**Sr**	-0.741	-0.741	0.938	0.001
**Dy**	0.372	0.372	0.930	0.001
**Ge**	0.434	0.434	0.926	0.001
**B**	0.309	0.309	0.924	0.001
**Si**	0.391	0.391	0.922	0.001
**Br**	-0.534	-0.534	0.916	0.001
**Sm**	0.376	0.376	0.914	0.001
**Ba**	0.433	0.433	0.910	0.001
**Cs**	0.378	0.378	0.903	0.001
**U**	-0.695	-0.695	0.903	0.002
**Rb**	0.358	0.358	0.901	0.001
**K**	0.227	0.227	0.898	0.001
**Gd**	0.381	0.381	0.898	0.001
**Li**	0.312	0.312	0.897	0.001
**Na**	-0.267	-0.267	0.896	0.001
**Mn**	0.376	0.376	0.895	0.001
**Fe**	0.383	0.383	0.887	0.001
**Mo**	0.528	0.528	0.886	0.003
**Al**	0.502	0.502	0.502	0.003
**V**	0.502	0.502	0.502	0.003
**Ga**	0.502	0.502	0.502	0.003
**Cd**	0.502	0.502	0.502	0.003
**Sb**	0.502	0.502	0.502	0.003
**Tl**	0.502	0.502	0.502	0.003
**Th**	0.502	0.502	0.502	0.003
**W**	0.5	0.5	0.5	0.002
**Y**	-0.79	-0.79	-0.79	0.001
**factor**	**CCA1**	**CCA2**	**r^2**	**Pr(>r)**
**Yb**	0.39	0.39	0.854	0.001

factor	CCA1	CCA2	r^2	Pr(>r)
**pH**	0.078	0.078	0.845	0.001
**La**	-0.325	-0.325	0.816	0.004
**Ho**	0.401	0.401	0.794	0.002
**Er**	0.401	0.401	0.792	0.003
**Ce**	0.199	0.199	0.756	0.005
**Pr**	0.419	0.419	0.683	0.011
**Nd**	0.145	0.145	0.583	0.033
**eH**	-0.967	-0.967	0.500	0.035
**Pb**	0.671	0.671	0.083	0.756
**Eu**	0.671	0.671	0.083	0.756
**P**	0	0	0.008	1
**Se**	0	0	0.008	1
**Hg.**	0	0	0.008	1
**Nb**	0	0	0.008	1
**Ru**	0	0	0.008	1
**Rh**	0	0	0.008	1
**Pd**	0	0	0.008	1
**Ag**	0	0	0.008	1
**Ir**	0	0	0.008	1
**Pt**	0	0	0.008	1
**Sc**	0	0	0	1
**Ti**	0	0	0	1
**Cr**	0	0	0	1
**Co**	0	0	0	1
**Ni**	0	0	0	1
**Cu**	0	0	0	1
**Te**	0	0	0	1
**Tb**	0	0	0	1
**Tm**	0	0	0	1
**Lu**	0	0	0	1
**Hf**	0	0	0	1
**Ta**	0	0	0	1
**Re**	0	0	0	1
**Os**	0	0	0	1
**Au**	0	0	0	1
**Bi**	0	0	0	1

Supplementary Table S5. Morphological and metabolic properties of closest cultivated relatives of most abundant taxa

ASV	Genus	Sampling site with maximum abundance	Sample type with maximal abundance	Max. abundance, %	manual NCBI/type material best BLAST hit (BBH)	Morphology (width / length in µm) of BBH*	T_{opt}, °C (min - max) of BBH*	Characteristic metabolic features of BBH*	Reference
**ASV_0002**	Thiofaba	Ursdon	0.45 µm filter	72.71	Thiofaba tepidiphila BDA453 (99.6%)	rods (0.4 / 0.5-0.9)	45 (20-51)	obligate autotroph; aerobe, oxidizes reduced sulfur compounds	[37]
**ASV_0003**	Unclassified Hydrogenophilaceae (Family)	Biragzang	0.45 µm filter	51.27	Annwoodia aquaesulis DSM 4255 (96.02%)	rods (0.3 / 0.9)	43 (30-55)	facultatively autotroph (via Calvin cycle); hetetotrophic growth with yeast extract; oxidizes reduced sulfur compounds with oxygen or nitrate as the electron acceptors	[34]
**ASV_0004**	Tepidimonas	Biragzang	microbial mat	26.34	Tepidimonas taiwanensis LMG 22826 (100%)	rods (0.4-1 / 0.8-2)	50-55 (25-65)	obligate heterotroph (uses of amino acids, organic acids and some sugars as carbon and energy sources); aerobe; oxidizes reduced sulfur compounds	[66]
**ASV_0005**	Acinetobacter	Karmadon 4138	0.45 µm filter	46.03	Acinetobacter johnsonii ATCC 17909 (99.6%)	rods (0.9-1.6 / 1.5-2.5)	~33-42 (ND)	obligate heterotroph (uses wide range of organic compunds); aerobe; oxidizes organic reducing equivalents via aerobic respiration	[69]

Supplementary Table S5. Morphological and metabolic properties of closest cultivated relatives of most abundant taxa (continued)

ASV	Genus	Sampling site with maximum abundance	Sample type with maximal abundance	Max. abundance, %	manual NCBI/type material best BLAST hit (BBH)	Morphology (width / length in µm) of BBH*	T_{opt}, °C (min - max) of BBH*	Characteristic metabolic features of BBH*	Reference
**ASV_0016**	Ahniella	Biragzang	microbial mat	17.76	Dokdonella fugitiva A3 (94.82%)	rods (0.4 / 1.7-5.8)	37-40 (15-50)	heterotroph; strict aerobe; utilizes various sugars and amino acids	[70]
**ASV_0007**	Klebsiella	Karmadon 4138	sediment	19.13	Enterobacteriaceae spp. (100%)	rods (0.3-1 / 0.6–6)	~30-37 (~5-44.5)	obligate heterotroph; facultative anaerobe; possess respiratory and fermentative type of metabolism	[71]
**ASV_0008**	Pseudomonas	Karmadon 4138	sediment	28.95	Pseudomonas khazarica TBZ2 (100%)	rods (0.5-1 / 1.5-5)	~28 (4-45)	obligate heterotroph; facultative anaerobe; possess aerobic respiration and nitrate reduction	[72]
**ASV_0010**	Tepidicella	Biragzang	0.22 µm filter	27.38	Tepidicella xavieri TU-16 (100%)	rods (0.5-1 / 1-2)	45 (25-55)	obligate heterotroph (uses of amino acids and organic acids as carbon source); aerobe; oxidizes reduced sulfur compounds	[65]
**ASV_0011**	Defluviimonas	Karmadon 4135	0.45 µm filter	17.89	Albidovulum inexpectatum FRR-10 (100%)	rods (0.4-0.6 / 1.4-2.2)	50 (ND-60)	obligate heterotroph (uses of amino acids, organic acids, sugars and polyols as carbon and energy sources); aerobe; possess aerobic respiration	[73]

Supplementary Table S5. Morphological and metabolic properties of closest cultivated relatives of most abundant taxa (continued)

ASV	Genus	Sampling site with maximum abundance	Sample type with maximal abundance	Max. abundance, %	manual NCBI/type material best BLAST hit (BBH)	Morphology (width / length in µm) of BBH*	T_{opt}, °C (min - max) of BBH*	Characteristic metabolic features of BBH*	Reference
**ASV_0012**	Thiobacter	Karmadon 4138	0.22 µm filter	26.57	Thiobacter subterraneus C55 (100%)	rods (0.4-0.5 / 1.1-1.9)	50-55 (35-62)	obligate autotroph; aerobe, oxidizes reduced sulfur compounds	[64]
**ASV_0014**	Ignavibacterium	Karmadon 4138	sediment	10.39	Ignavibacterium album JCM 16511 (99.6%)	rods (0.2-0.3 / 2-16)	45 (30-55)	faculatatively anaerobic heterothroph, capable to utilize various organic substrates	[74]
**ASV_0021**	Candidatus Alysiosphaera	Karmadon 4135	0.45 µm filter	5.84	Arboricoccus pini B29T1 (91.63%)	diplococci (0.4-0.5 / 0.9-1.2)	26-30 (15-37)	obligate heterotroph (utilizes sugars and some organic acids); facultative anaerobe; possess aerobic respiration	[75]
**ASV_0022**	Schleiferia	Biragzang	microbial mat	27.07	Schleiferia thermophila TU-20 (98.8%)	rods (0.5-0.7 / 0.5-1.5)	50 (25-65)	heterotroph; strict aerobe; proteinolytic; capable of denitrification (nitrate to N2)	[76]
**ASV_0023**	Unclassified Alphaproteobacteria (Class)	Karmadon 4135	sediment	7.86	Iodidimonas spp. (<93.2%)	rods (0.3-1.1 / 1.2-4.4)	30 (4-40)	obligate heterotroph (utilizes sugars and YE); aerobe; possess aerobic respiration	[77]
**ASV_0024**	Unclassified SR-FBR-L83 (Family)	Karmadon 4138	sediment	8.73	Melioribacter roseus P3M-2 (92.4%)	rods (0.25-0.35 / 0.65-10)	55 (35-60)	obligate heterotroph (utilizes proteinaceous compounds, simple and complex carbohydrates); facultative anaerobe; capable of aerobic and anaerobic respiration and fermentation	[74]

Supplementary Table S5. Morphological and metabolic properties of closest cultivated relatives of most abundant taxa (continued)

ASV	Genus	Sampling site with maximum abundance	Sample type with maximal abundance	Max. abundance, %	manual NCBI/type material best BLAST hit (BBH)	Morphology (width / length in µm) of BBH*	T_{opt}, °C (min - max) of BBH*	Characteristic metabolic features of BBH*	Reference
**ASV_0026**	Raineya	Biragzang	sediment	8.40	Raineya orbicola SPSPC-11 (92.8%)	rods or filaments (0.5-0.8 / 5-15)	50 (35-60)	obligate heterotroph (utilizes proteins, amino acids and organic acids); aerobe; possess aerobic respiration	[78]
**ASV_0027**	Unclassified Saprospiraceae (Family)	Biragzang	sediment	8.21	Phaeodactylibacter xiamenensis KD52 (92.4%)	rods (0.5-0.6 / 5-10.7)	28 (20-37)	obligate heterotroph; aerobe; possess aerobic respiration	[79]
**ASV_0029**	Phaeodactylibacter	Karmadon 4135	sediment	7.80	Saprospiraceae spp. (<89.3%)	rods or filaments (0.5-1.4 / 2.4-21.2)	~20-28 (3-37)	obligate heterotroph; aerobe; possess aerobic respiration	[79]
**ASV_0031**	Porphyrobacter	Biragzang	sediment	7.18	Porphyrobacter spp. (100%)	rods (0.5-1.0 / 0.5-2.8)	30-37 (10-60)	aerobic chemoheterotroph, utilizing glucose and peptides mixtures	[69]
**ASV_0032**	Blvii28 wastewater-sludge group	Ursdon	sediment	11.21	Tenuifilum thalassicum 38H-str (98%)	rods or filaments (0.11-0.17 / 3-10)	50 (20-65)	obligate heterotroph (utilizes proteins and carbohydrates); anaerobe; capable of sulfur reduction; possess fermentative metabolism	[63]
**ASV_0034**	Enhydrobacter	Karmadon 4138	0.45 µm filter	6.12	Moraxella osloensis NCTC 10465 (100%)	rods (1-2 / 1-5) or cocci (0.6–1)	33-37 (ND)	obligate heterotroph; aerobe; possess aerobic respiration	[80]

Supplementary Table S5. Morphological and metabolic properties of closest cultivated relatives of most abundant taxa (continued)

ASV	Genus	Sampling site with maximum abundance	Sample type with maximal abundance	Max. abundance, %	manual NCBI/type material best BLAST hit (BBH)	Morphology (width / length in µm) of BBH*	T_{opt}, °C (min - max) of BBH*	Characteristic metabolic features of BBH*	Reference
**ASV_0035**	Thermodesulfitimonas	Ursdon	0.22 µm filter	7.66	Thermodesulfitimonas autotrophica SF97 (96.05%)	rods (0.5-0.6 / 1-2)	65 (45-72)	anaerobe, sulfite reducing (with H2 as an elecron donor), autotroph (via transaldolase Calvin cycle variant or Wood-Ljungdahl pathway)	[81]
**ASV_0037**	Micrococcus	Karmadon 4138	0.45 µm filter	5.78	Micrococcus luteus NCTC 2665 (100%)	cocci (0.5-2)	37 (ND)	obligate heterotroph; aerobe; possess aerobic respiration	[82]
**ASV_0040**	Meiothermus	Biragzang	microbial mat	6.97	Meiothermus ruber DSM 1279 (100%)	rods (0.5-0.8 / 3-6)	60 (35-70)	obligate heterotroph (utilizes sugars and organic acids); aerobe; possess aerobic respiration	[83]
**ASV_0041**	Sulfurospirillum	Ursdon	sediment	9.55	Sulfurospirillum deleyianum DSM 6946 (100%)	vibrions or spirills (0.3-0.5 / 1-3)	ND (20-42)	facultative autotroph; anaerobe/microaerobe; possess aerobic respiration with organic acids as the electron donor or anaerobic respiration with hydrogen or formate as the electron donor and nitrate, nitrite, sulfite, thiosulfate, elemental sulfur, DMSO or fumarate as the electron acceptor	[84]

Supplementary Table S5. Morphological and metabolic properties of closest cultivated relatives of most abundant taxa (continued)

ASV	Genus	Sampling site with maximum abundance	Sample type with maximal abundance	Max. abundance, %	manual NCBI/type material best BLAST hit (BBH)	Morphology (width / length in µm) of BBH*	T_{opt}, °C (min - max) of BBH*	Characteristic metabolic features of BBH*	Reference
**ASV_0043**	Unclassified Thermodesulfovibrionia (Class)	Biragzang	0.22 µm filter	5.84	Dissulfurispira thermophila T55J (98.4%)	rods or spirals (0.32-0.49 / 0.5-19)	53-57 (25-60)	obligate autotroph; anaerobe; possess disproportionation of thiosulfate and elemental sulfur,	[85]
**ASV_0044**	Dissulfurimicrobium	Ursdon	0.45 µm filter	5.27	Dissulfurirhabdus thermomarina SH388 (94.4%)	rods (0.2-0.4 / 1-2.5)	50 (25-58)	anaerobe, sulfite reducing (with H2 as an elecron donor), autotroph. Able to grow by disproportionation of sulfite and elemental sulfur into sulfide and sulfate	[86]
**ASV_0047**	Candidatus Nitrososphaera	Karmadon 4135	sediment	6.22	Nitrososphaera viennensis EN76 (98.4%)	cocci (0.65-0.91)	42 (28-47)	mixotroph; aerobe; oxidizes ammonia to nitrite with oxygen as the electron acceptor	[87]
**ASV_0048**	Unclassified Spirochaetaceae (Family)	Ursdon	0.22 µm filter	5.00	Rectinema cohabitans HM (100%)	rods (0.3-0.5 / 1-2.5)	37 (12-50)	obligate heterotroph; anaerobe; ferments various sugars	[88]
**ASV_0068**	Treponema	Ursdon	sediment	5.28	Treponema caldarium DSM 7334 (100%)	helicals (0.2-0.3 / 15-45)	48-52 (25-60)	obligate heterotroph; anaerobe; ferments carbohydrates	[89]

Supplementary Table S6. Results of BLAST search of the most abundant deep uncultured lineages detected in North Ossetian thermal habitats.

ASV number	Silva138 predicted taxonomy	NCBI accession of BLAST hit	Isolation source	Geolocation	Identity, %
**ASV_0084**	Unclassified TSAC18 (Class)	KP930849.1	post-volcanic pyroclastic surface	Kasatochi Island, Alaska, USA	96.81
		FJ936877.1	volcano mud	Avachinsky volcano, Kamtchatka, Russia	96.02
		DQ088766.1	Fracture-derived groundwater	South Africa	94.02
		EF562164.1	Henderson Molybdenum Mine	USA: Colorado, Empire	93.63
		MH387930.1	Thermogenic travertine	Greece	92.46
**ASV0001**	Unclassified Firmicutes (Phylum)	KP930849.1	soil or post-volcanic pyroclastic surface	Kasatochi Island, Alaska, US	96.02
		FJ936877.1	volcano mud	Avachinsky, Kamtchatka, Russia	93.23
		EF562164.1	Henderson Molybdenum Mine	USA: Colorado, Empire	92.06
		DQ088766.1	Fracture-derived groundwater	South Africa	91.63
		MN157828.1	anaerobic digester	n/d	91.16
**ASV_0024**	Unclassified SR-FBR-L83 (Family)	KM700928.1	hot spring	Yellowstone National Park, US	99.20
		AF027013.1	Obsidian Pool sediment	Yellowstone National Park, US	97.61
		MF612776.1	acidogenic reactor	n/d	97.21
		MF894809.1	coal seam environment	Australia	97.55
		MN156931.1	anaerobic digester	n/d	96.02
**ASV_0051**	Unclassified Parcubacteria	LC070984.1	deep sea mud	Japan, Sagami bay	92.71
		AB825243.1	sediment	Japan:Okinawa, Okinawa Trough, Iheya North Knoll	92.28
		AB177199.1	hydrate bearing subseafloor sediment	Peru	92.31
		AB827012.1	hadopelagic sediment	Japan, Ogasawara Trench	91.50
		HF545524.1	deep sediment	Mexico:Sonora Margin, Guaymas Basin	91.53
**ASV_0054**	Candidatus Omnitrophus	KY517874.1	lake water	Canada: Experimental Lakes Area, Ontario	94.02
		KF886090.1	hydrothermal carbonate chimney	New Caledonia: Prony Bay	92.43
		AB924396.1	deep groundwater	Japan:Shizuoka, Yaizu	90.44
		KC604650.1	groundwater	Mahomet aquifer of east-central Illinois, US	89.72
		KU090195.1	oil field	n/d	89.24

Supplementary Table S7. Sequence and design scheme of primers, used for 16S community profiling.

16S GENE PRIMERS

Primer name	Target region	Illumina partial adapter sequence	Pad and hetero-geneity spacer	16S rRNA gene annealing sequence	Full sequence 5' - 3'
V4_515F	V4	TCGTCGGCAGCGTCAGATGTGTATAAGAGACAG	NN	GTGBCAGCMGCCGCGGTAA	TCGTCGGCAGCGTCAGATGTGTATAAGAGACAGNNGTGBCAGCMGCCGCGGTAA
V4_515F	V4	TCGTCGGCAGCGTCAGATGTGTATAAGAGACAG	NNN	GTGBCAGCMGCCGCGGTAA	TCGTCGGCAGCGTCAGATGTGTATAAGAGACAGNNNGTGBCAGCMGCCGCGGTAA
V4_515F	V4	TCGTCGGCAGCGTCAGATGTGTATAAGAGACAG	NNNN	GTGBCAGCMGCCGCGGTAA	TCGTCGGCAGCGTCAGATGTGTATAAGAGACAGNNNNGTGBCAGCMGCCGCGGTAA
V4_515F	V4	TCGTCGGCAGCGTCAGATGTGTATAAGAGACAG	NNNNN	GTGBCAGCMGCCGCGGTAA	TCGTCGGCAGCGTCAGATGTGTATAAGAGACAGNNNNNGTGBCAGCMGCCGCGGTAA
V4_806R	V4	GTCTCGTGGGCTCGGAGATGTGTATAAGAGACAG	NN	GACTACNVGGGTMTCTAATCC	GTCTCGTGGGCTCGGAGATGTGTATAAGAGACAGNNGACTACNVGGGTMTCTAATCC
V4_806R	V4	GTCTCGTGGGCTCGGAGATGTGTATAAGAGACAG	NNN	GACTACNVGGGTMTCTAATCC	GTCTCGTGGGCTCGGAGATGTGTATAAGAGACAGNNNGACTACNVGGGTMTCTAATCC
V4_806R	V4	GTCTCGTGGGCTCGGAGATGTGTATAAGAGACAG	NNNN	GACTACNVGGGTMTCTAATCC	GTCTCGTGGGCTCGGAGATGTGTATAAGAGACAGNNNNGACTACNVGGGTMTCTAATCC
V4_806R	V4	GTCTCGTGGGCTCGGAGATGTGTATAAGAGACAG	NNNNN	GACTACNVGGGTMTCTAATCC	GTCTCGTGGGCTCGGAGATGTGTATAAGAGACAGNNNNNGACTACNVGGGTMTCTAATCC

ILLUMINA P5 INDEXING PRIMERS

Primer name	index 2 read in sample sheet	Illumina P5 adapter	i5 index (index 2)	Illumina adapter sequence	Full sequence 5' - 3'
R1TM25	ACTGAT	AATGATACGGCGACCACCGAGATCTACAC	ACTGAT	TCGTCGGCAGCGTC	AATGATACGGCGACCACCGAGATCTACACACTGATTCGTCGGCAGCGTC
R1TM26	ATGAGC	AATGATACGGCGACCACCGAGATCTACAC	ATGAGC	TCGTCGGCAGCGTC	AATGATACGGCGACCACCGAGATCTACACATGAGCTCGTCGGCAGCGTC
R1TM27	ATTCCT	AATGATACGGCGACCACCGAGATCTACAC	ATTCCT	TCGTCGGCAGCGTC	AATGATACGGCGACCACCGAGATCTACACATTCCTTCGTCGGCAGCGTC
R1TM28	CAAAAG	AATGATACGGCGACCACCGAGATCTACAC	CAAAAG	TCGTCGGCAGCGTC	AATGATACGGCGACCACCGAGATCTACACCAAAAGTCGTCGGCAGCGTC
R1TM29	CAACTA	AATGATACGGCGACCACCGAGATCTACAC	CAACTA	TCGTCGGCAGCGTC	AATGATACGGCGACCACCGAGATCTACACCAACTATCGTCGGCAGCGTC
R1TM30	CACCGG	AATGATACGGCGACCACCGAGATCTACAC	CACCGG	TCGTCGGCAGCGTC	AATGATACGGCGACCACCGAGATCTACACCACCGGTCGTCGGCAGCGTC
R1TM31	CACGAT	AATGATACGGCGACCACCGAGATCTACAC	CACGAT	TCGTCGGCAGCGTC	AATGATACGGCGACCACCGAGATCTACACCACGATTCGTCGGCAGCGTC
R1TM32	CACTCA	AATGATACGGCGACCACCGAGATCTACAC	CACTCA	TCGTCGGCAGCGTC	AATGATACGGCGACCACCGAGATCTACACCACTCATCGTCGGCAGCGTC

Supplementary Table S7. Sequence and design scheme of primers, used for 16S community profiling. (continued)

ILLUMINA P7 INDEXING PRIMERS

Primer name	index 1 read in sample sheet	Illumina P7 adapter	i7 index (index 1)	Illumina adapter sequence	Full sequence 5' - 3'
R2TM51	TGAAGG	CAAGCAGAAGACGGCATACGAGAT	CCTTCA	GTCTCGTGGGCTCGG	CAAGCAGAAGACGGCATACGAGATCCTTCAGTCTCGTGGGCTCGG
R2TM52	AACATA	CAAGCAGAAGACGGCATACGAGAT	TATGTT	GTCTCGTGGGCTCGG	CAAGCAGAAGACGGCATACGAGATTATGTTGTCTCGTGGGCTCGG
R2TM53	CGCGTC	CAAGCAGAAGACGGCATACGAGAT	GACGCG	GTCTCGTGGGCTCGG	CAAGCAGAAGACGGCATACGAGATGACGCGGTCTCGTGGGCTCGG
R2TM54	GATACA	CAAGCAGAAGACGGCATACGAGAT	TGTATC	GTCTCGTGGGCTCGG	CAAGCAGAAGACGGCATACGAGATTGTATCGTCTCGTGGGCTCGG
R2TM55	GGTGTG	CAAGCAGAAGACGGCATACGAGAT	CACACC	GTCTCGTGGGCTCGG	CAAGCAGAAGACGGCATACGAGATCACACCGTCTCGTGGGCTCGG
R2TM56	TAAGAA	CAAGCAGAAGACGGCATACGAGAT	TTCTTA	GTCTCGTGGGCTCGG	CAAGCAGAAGACGGCATACGAGATTTCTTAGTCTCGTGGGCTCGG
R2TM57	AGCGAG	CAAGCAGAAGACGGCATACGAGAT	CTCGCT	GTCTCGTGGGCTCGG	CAAGCAGAAGACGGCATACGAGATCTCGCTGTCTCGTGGGCTCGG
R2TM58	CGGTTA	CAAGCAGAAGACGGCATACGAGAT	TAACCG	GTCTCGTGGGCTCGG	CAAGCAGAAGACGGCATACGAGATTAACCGGTCTCGTGGGCTCGG

SUPPLEMENTARY FIGURES

Supplementary Figure S1. PCA ordination of sampling sites according to environmental parameters.

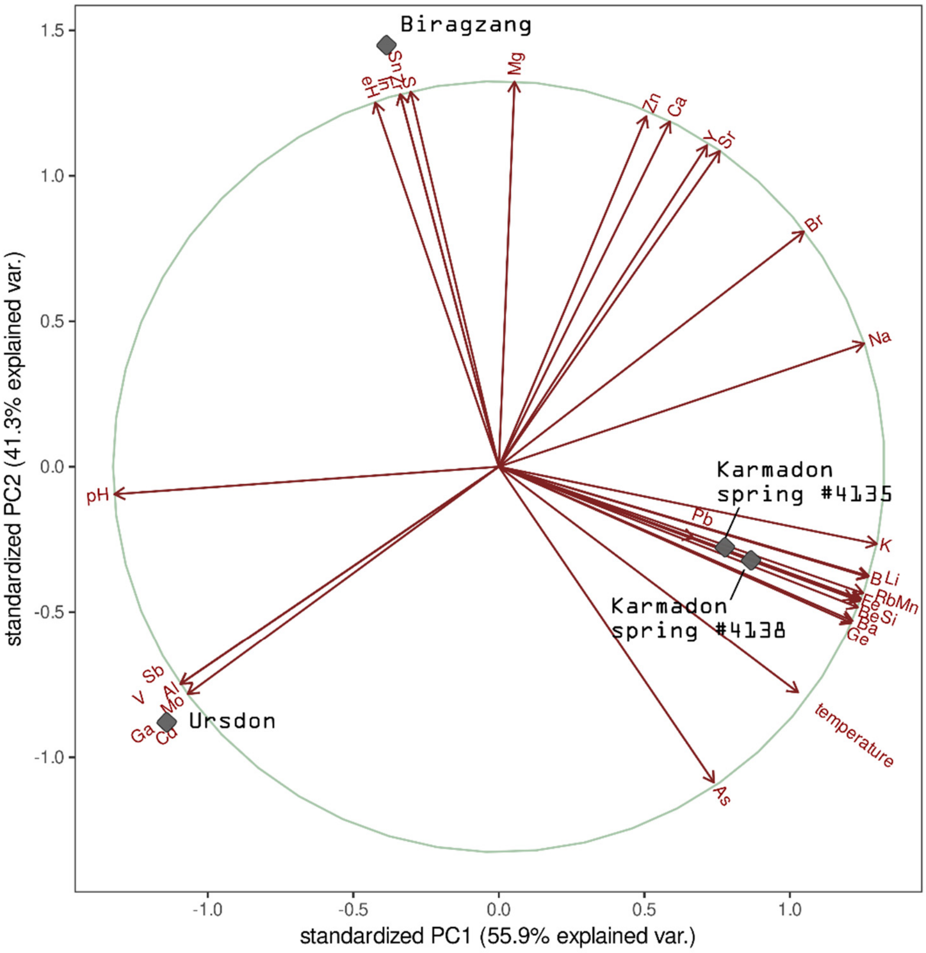

Supplementary Figure S2. NMDS analysis of repeatability of PCR replicates

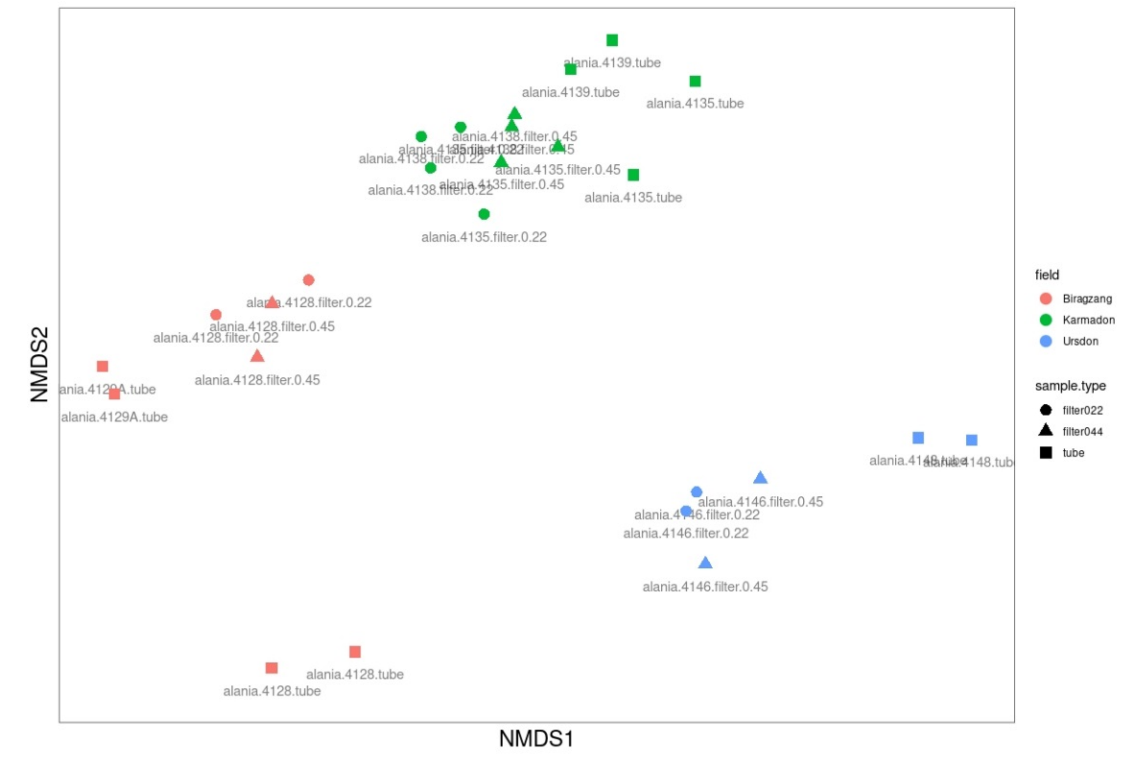

Supplementary Figure S3. Rarefaction curves of North-Ossetian thermophilic microbial communities.

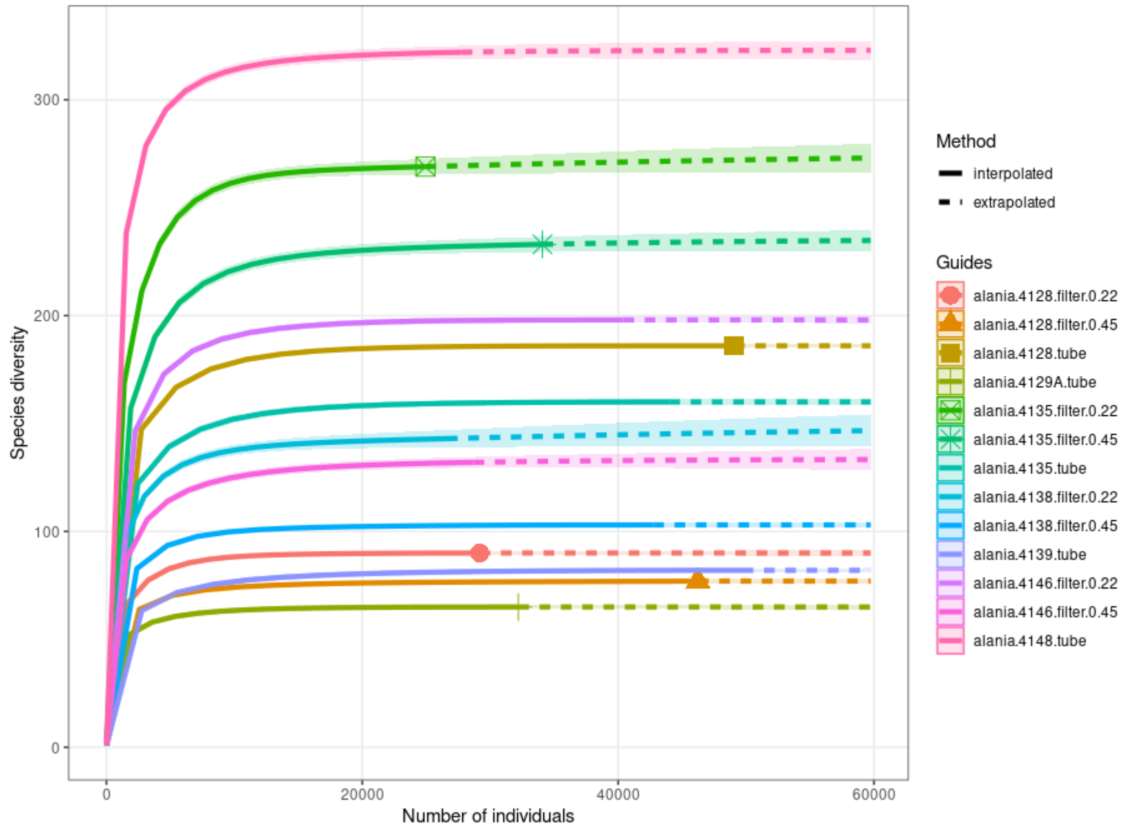

Supplementary Figure S4. Bubble plot of taxonomic composition of sampled microbial communities (by order)

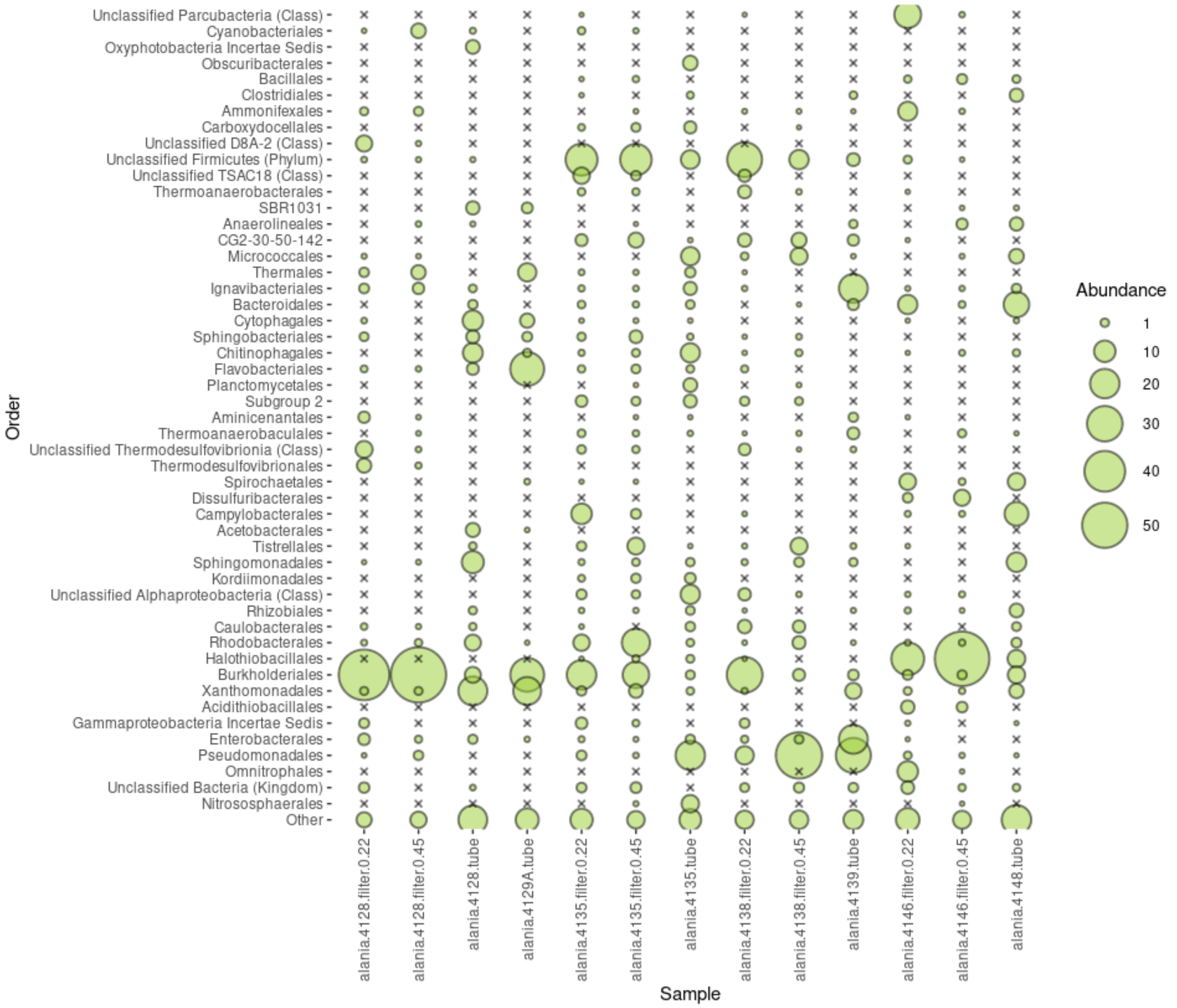

Supplement: Supplementary file 1 [file biology-10-01352-s001.zip › biology-1495147-supplementary.pdf]
